# Isotope Effect in D_2_O Negative Ion Formation in Electron Transfer Experiments: DO–D Bond Dissociation Energy

**DOI:** 10.1021/acs.jpclett.3c00786

**Published:** 2023-06-05

**Authors:** Sarvesh Kumar, Masamitsu Hoshino, Boutheïna Kerkeni, Gustavo García, Paulo Limão-Vieira

**Affiliations:** † Atomic and Molecular Collisions Laboratory, CEFITEC, Department of Physics, Universidade NOVA de Lisboa, 2829-516 Caparica, Portugal; ‡ Department of Materials and Life Sciences, Sophia University, Tokyo 102-8554, Japan; § ISAMM, Université de la Manouba, La Manouba 2010, Tunisia; ∥ Département de Physique, LPMC, Faculté des Sciences de Tunis, Université de Tunis el Manar, Tunis 2092, Tunisia; ⊥ Instituto de Física Fundamental, Consejo Superior de Investigaciones Científicas (CSIC), Serrano 113-bis, 28006 Madrid, Spain

## Abstract

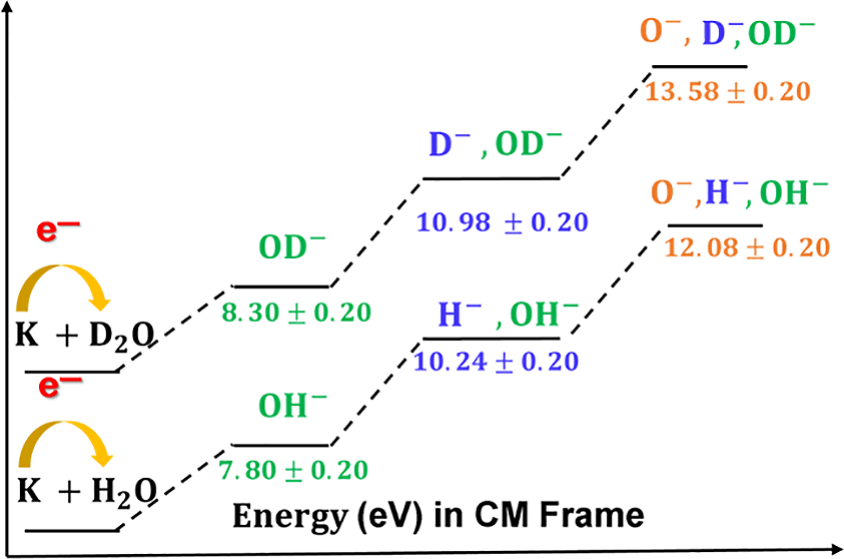

H_2_O/D_2_O negative ion time-of-flight mass spectra from electron transfer processes at different collision energies with neutral potassium yield OH^–^/OD^–^, O^–^, and H^–^/D^–^. The branching ratios show a relevant energy dependence with an important isotope effect in D_2_O. Electronic state spectroscopy of water has been further investigated by recording potassium cation energy loss spectra in the forward scattering direction at an impact energy of 205 eV (lab frame), with quantum chemical calculations for the lowest-lying unoccupied molecular orbitals in the presence of a potassium atom supporting most of the experimental findings. The DO–D bond dissociation energy has been determined for the first time to be 5.41 ± 0.10 eV. The collision dynamics revealed the character of the singly excited (1b_2_^–1^) molecular orbital and doubly excited states in such K–H_2_O and K–D_2_O collisions.

Electron-induced processes in biologically relevant molecules have been central to the assessment of the underlying molecular mechanisms responsible for bond excision and chemical modification after interaction of primary radiation with living tissue.^1^ The seminal work of Sanche and co-workers^2^ has shown that low-energy electron-initiated reactions cause structural DNA modifications via single- and double-strand breaks (SSBs and DSBs, respectively), where the quantum yields for such degradation processes are reminiscent of a resonant behavior with electron energy. The role of water molecules in the cellular environment has been shown to be pivotal in determining the biological damage imparted on a cell by free radical formation upon water radiolysis; however, a large portion of the damage might be due to low-energy electron processes.^3^ Wang et al.^4^ have shown the role of dissociative electron transfer reactions of presolvated electrons with DNA nucleotides, where reduction processes yielding SSBs and DSBs are prevalent mechanisms in aqueous solutions. The appropriateness of water as an underlying molecular constituent for describing radiation damage in living tissue on charged-particle transport mechanisms^5,6^ has also been addressed. Moreover, particle track simulations in gaseous and liquid water produced by electrons^7−9^ and positrons^10^ (0.1–10000 eV), particle track simulations with proton impact^11^ and photon interactions with H_2_O providing detailed information about secondary electron tracks, energy deposition, and interaction processes at the molecular level have also been reported.^12^

The negative ion formation of water bare molecules has been attracting the attention of the international scientific community for at least 90 years,^13^ though we still note a global interest in investigating the electronic state spectroscopy of its anionic states. Dissociative electron attachment (DEA) to H_2_O has been reported on several occasions by experimental^14−27^ and theoretical methodologies,^17,28−38^ although a global consensus about the nuclear dynamics governing the lowest-energy Feshbach resonances of H^–^ and O^–^ has not yet been reached.^39,40^ Additionally, the anionic fragments 1u, 16u, and 17u were reported from electron transfer experiments in high-energy (1–4 keV) collisions of H^–^, O^–^, and OH^–^ with water molecules.^41^ Regardless, theoretical calculations related to water ^2^B_1_, ^2^A_1_, and ^2^B_2_ Feshbach resonances have been employed to obtain cross sections for DEA^29,30,35^ and the potential energy surfaces of such metastable states.^36−38,42^ Electron scattering^7−9,43−45^ and ion scattering,^46^ and electronic excitation^5,6,13,47−49^ in single water molecules and aggregates,^50^ have been reported, while H_2_O bond dissociation energies have been determined by experimental^23^ and theoretical methods.^32^ In the unimolecular decomposition of the temporary negative ion formed after electron capture, the sort of fragmentation and the relative yields that can be attained in electron transfer processes may differ from those of DEA experiments. The collision dynamics in electron transfer processes mediated by the crossing of covalent and ionic potential energy curves (and/or surfaces) involving the atomic projectile and the molecular target is different from that of a free electron attachment process^51−55^ (Introductory Note in the Supporting Information). At the radiobiology level, the most prevalent processes are related to electron transfer rather than electron impact. Thus, apart from single-electron interaction, investigating molecular damage must be complemented with electron capture of “bound” electrons (like those provided in atom–molecule collisions).^56^

The electronic ground state valence configuration of water is (1a_1_)^2^ (2a_1_)^2^ (1b_2_)^2^ (3a_1_)^2^ (1b_1_)^2^: X ~^1^A_1_, where the outermost orbitals (1b_1_, 3a_1_, and 1b_2_) have n_O_(2p_*x*⊥HOH_) out-of-plane, n̅_O_(2p_*z*∥HOH_) in-plane, and weakly σ_OH_ and 2p_*y*_ σ_OH_ character, respectively. The time-of-flight (TOF) mass spectra of the different anions formed during electron transfer from potassium collisions with H_2_O and D_2_O (Experimental Method in the Supporting Information) were obtained in the energy ranges of 9.2–198.9 and 9.8–213.6 eV in the center-of-mass (CM) frame. In this collision energy range, fragmentation of H_2_O yields OH^–^, O^–^, and H^–^, while that of D_2_O results in OD^–^, O^–^, and D^–^, with no evidence of parent anion formation from both molecules. The branching ratios (BRs) for the fragment anions from these molecules are shown in Figure 1, with the most abundant anions across the entire collision energy range investigated assigned to OH^–^/OD^–^.

**Figure 1:**
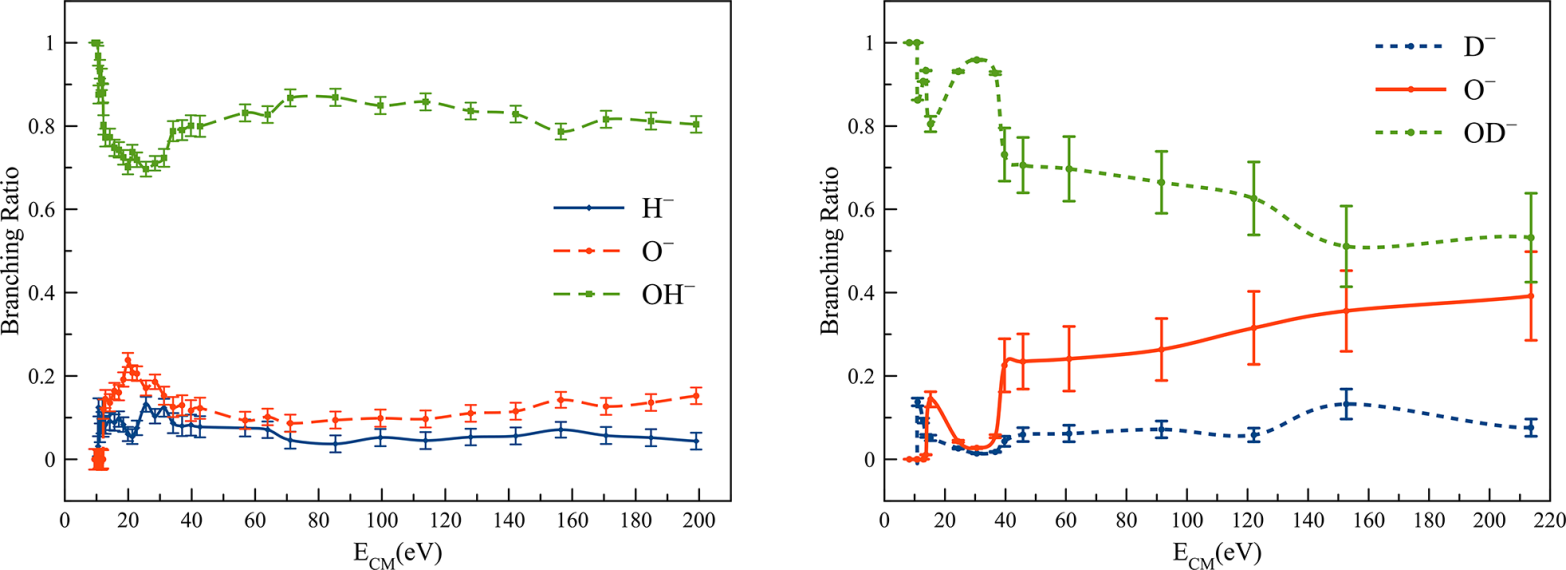
H_2_O and D_2_O BRs of the anions formed as a function of the collision energy in the center-of-mass frame. Error bars are related to the experimental uncertainty associated with the ion yields. The dashed and solid lines were added just to guide the eye.

In the case of H_2_O (and D_2_O), the BRs show a strong energy dependence up to *E*_CM_ ∼ 30 eV; beyond this value, the yields are almost insensitive to the collision energy within experimental uncertainty. Above this energy, the most intense fragment anion amounting to >80% of the total anion yield has been assigned to OH^–^, followed by O^–^ and H^–^. With respect to D_2_O, up to *E*_CM_ = 40 eV OD^–^ accounts for >70% of the total anion yield and together with O^–^ shows a modest energy dependence from 40 to 150 eV, remaining almost constant at higher energies. Another relevant aspect of D_2_O BRs pertains to the contribution of the monoanions above 40 eV, surpassing together >30% of the total anion yield. The collision energy dependence of the D_2_O fragment anions relative to H_2_O renders a strong isotope effect for the former molecule.

In contrast, dissociative electron attachment experiments up to ∼9 eV show that H^–^/D^–^ fragment anions are found to be the most abundant, followed by O^–^ and OH^–^/OD^–^.^16,27^ These anions are formed through three transient anion states, with broad features peaking at 6.5 (7.0), 8.6 (9.0) and 11.8 (12.0) eV (see Table S1), and assigned to core excited Feshbach resonances with electron configurations of (1b_1_^–1^4a_1_^2^) ^2^B_1_, (3a_1_^–1^4a_1_^2^) ^2^A_1_, and (1b_2_^–1^4a_1_^2^) ^2^B_2_,^17^ the former two correlating with the parent Rydberg states in the vacuum ultraviolet spectrum of H_2_O at 7.464 and 9.991 eV.^57^

Figure 2 depicts the potassium cation (K^+^) energy loss spectra in the forward scattering direction (θ ≈ 0°) for K + H_2_O and K + D_2_O at *E*_CM_ = 58.3 and 62.5 eV. The experimental data have been smoothed and fitted with Gaussian functions to decompose the energy loss spectra, with vertical electron affinities and assignment of the most representative molecular orbitals (MOs) in Table 1.

**Figure 2:**
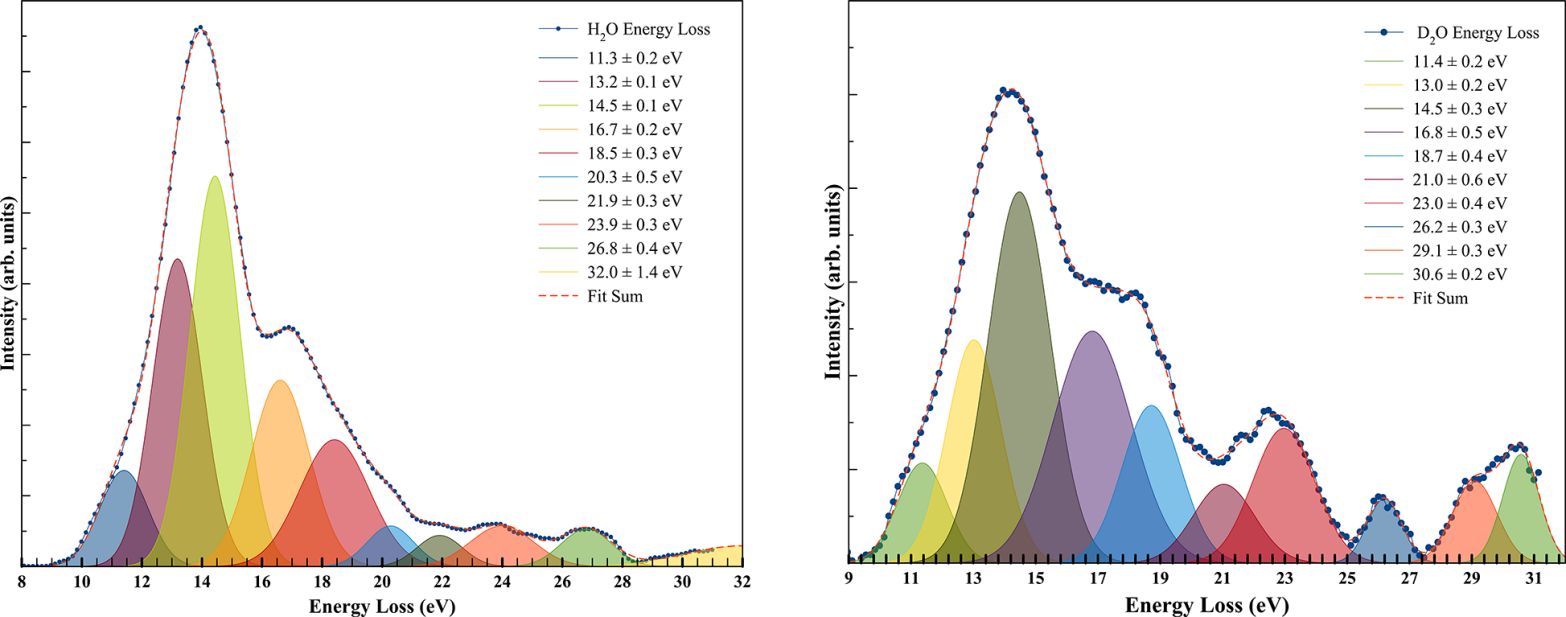
Energy loss spectra of K^+^ in the forward scattering direction (θ ≈ 0°) at impact energies of 58.3 eV for K + H_2_O and 62.5 eV for K + D_2_O in the center-of-mass frame. The uncertainty of the peaks results from the Gaussian fitting procedure.

**Table 1:** Assignment of Different Features from Gaussian Fittings to K^+^ Energy Loss Spectra from K + H_2_O and K + D_2_O Collisions at 58.3 and 62.5 eV^a^

K^+^ energy loss feature		vertical electron affinity				DEA resonances^1^
H_2_O	D_2_O	H_2_O	D_2_O	calculated vertical energy of MOs	assignment^b^	H_2_O and D_2_O
11.3 ± 0.2	11.4 ± 0.2	–6.96 ± 0.20	–7.06 ± 0.20	6.21 (LUMO+37); 7.85 (LUMO+38)	σ_OH/OD_^*^, σ_O–H/O–D_^*^, ^2^B_1_	6.5–7.0 (H^–^/D^–^, OH^–^/OD^–^)
13.2 ± 0.1	13.0 ± 0.2	–8.86 ± 0.10	–8.66 ± 0.20	8.94 (LUMO+40); 9.44 (LUMO+44)	σ_OH/OD_^*^, σ_O–H/O–D_^*^, ^2^A_1_	8.5–9.0 (H^–^, O^–^, OH^–^/OD^–^)
14.5 ± 0.1	14.5 ± 0.3	–10.16 ± 0.10	–10.16 ± 0.30	10.70 (LUMO+45)	σ_O–D_^*^, ^2^A_1_; *σ*_OH/OD_^*^, ^2^B_2_	∼9.0 (D^–^), ∼11.0 (OH^–^/OD^–^)
16.7 ± 0.2	16.8 ± 0.5	–12.36 ± 0.20	–12.46 ± 0.50	11.70 (LUMO+46)	σ_O–H/O–D_^*^, σ_OH/OD_^*^, ^2^B_2_	11.5–12.0 (H^–^/D^–^, O^–^, OH^–^/OD^–^)
18.5 ± 0.3	18.7 ± 0.4	–14.16 ± 0.30	–14.36 ± 0.40	13.60 (LUMO+47); 13.70 (LUMO+48)	σ_O–H/O–D_^*^, σ_OH/OD_^*^^c^	–
20.3 ± 0.5	21.0 ± 0.6	–15.96 ± 0.50	–16.66 ± 0.60	15.72 (LUMO+49); 16.65 (LUMO+50)	σ_OH/OD_^*^^c^	–
21.9 ± 0.3	23.0 ± 0.4	–17.56 ± 0.30	–18.86 ± 0.40	16.65 (LUMO+50); 19.61 (LUMO+51)	σ_O–H/O–D_^*^, σ_OH/OD_^*^^c^,^d^	–
23.9 ± 0.3	26.2 ± 0.3	–19.56 ± 0.30	–21.86 ± 0.30	20.47 (LUMO+52); 22.46 (LUMO+54)	singly excited (1b_2_^–1^) MO states	–
26.8 ± 0.4	29.1 ± 0.3	–22.46 ± 0.40	–24.76 ± 0.30	22.46 (LUMO+54); 25.26 (LUMO+56)	doubly excited (D1)	–
32.0 ± 1.4	30.6 ± 0.2	–27.66 ± 1.40	–26.26 ± 0.20	27.47 (LUMO+57)	doubly excited (D2)	–

a The uncertainties result from the Gaussian fitting procedure (values in electronvolts).

b See Table S1.

c Also σ_OH_/σ_OD_ → (*n* + 1)/(*n* + 2)s, σ_OH_/σ_OD_ → (*n* + 1)/(*n* + 2)p, and σ_OH_/σ_OD_ → (*n* + 1)/(*n* + 2)d.

d Singly excited (1b_2_^–1^) MO states.

The energy loss required to access a molecule’s electronic state, Δ*E*, is given by the difference between the ionization energy of the potassium atom, IE(K), and the electron affinity of that state at its maximum intensity, EA(*I*_max_), as Δ*E* = IE(K) – EA(*I*_max_).^58^ A close inspection of Figure 2 shows that the energy loss peaks of H_2_O and D_2_O have maximum intensities (*I*_max_) at 13.95 ± 0.30 eV, resulting in vertical electron affinities of −9.61 ± 0.3 eV, closely related to the broad DEA resonance features at ∼9 eV^14−16,18−20,23,25−27^ (Table 1).

*Ab initio* calculations have been performed to help with the assignment of the main relevant MOs involved in the electron transfer process (see Theoretical Method in the Supporting Information and Figures S1 and S2).

The BRs clearly show that for ∼10.1 and ∼10.8 eV (for H_2_O and D_2_O), OH^–^/OD^–^ ions are the only fragment ions formed. The reasonable explanation for the striking difference from DEA experiments (H^–^ dominates for the two lowest resonances at ∼6.5 and ∼8.6 eV) pertains to the role K^+^ formed after electron transfer, where a relevant Coulomb interaction may effectively stabilize the temporary negative ion (TNI), resulting in effective intramolecular processes that may allow the lowest-energy reactions to evolve. This is in assertion with the obtained experimental threshold energy values from TOF mass spectrometry data for the different exit channels in water negative ion formation (Table S2). It is interesting that in the presence of the potassium atom the water HOMO–LUMO energy difference has been calculated to be 2.7 eV (see Table S3), whereas in the bare molecule, it is 6.3 eV.^59^ Such an energy shift effect has been reported on several occasions and is due to the polarization induced by the presence of the potassium atom in the vicinity of the molecular target.^55,60−66^ Moreover, the strong σ_OH_^*^ antibonding character and the somewhat higher electron affinity of OH/OD (∼1.83 eV) relative to that of H/D (∼0.75 eV) (Table S4) may then dictate the collision-induced dissociation of water yielding OH^–^/OD^–^. Although there is some variation in the yields, the yields of OH^–^/OD^–^ decrease to ∼70% with increase in energy to ∼30–40 eV, while other dissociation channels are open, viz., O^–^ and H^–^/D^–^ formation.

The strong OH antibonding character of the 3sa_1_ orbital lends support to the idea that OH^–^ formation is the most intense dissociation channel, which in DEA experiments yields H^–^ as the dominant fragment ion formed via the ^2^B_1_ and ^2^A_1_ resonance states.^14−16,18−27,29,30,33,35,38^ The calculations of Haxton et al.^29^ predicted a lifetime of ∼110 fs for the ^2^B_1_ state, and within the framework of nuclear dynamics, autodetachment plays a minor role relative to TNI dissociation.^29^ The quasi-constant OH^–^ yield above 30 eV [∼80% (Figure 1)], with OD^–^ showing a similar tendency (within the experimental uncertainty) albeit with a lower yield (∼65%), results from the fast collision regime attained at those energies (≲40 fs), where K^+^ can no longer effectively stabilize the TNI. Thus, any electronic transition at these collision energies is mostly driven by a relevant antibonding character of the potential energy surface above the ground state in the Franck–Condon region.

The MO densities in Figure S2 that may contribute to the energy loss features with vertical values of −6.96 ± 0.10 and −7.06 ± 0.20 eV (Figure 2 and Table 1) are assigned to electron transfer from the potassium atom to H_2_O (and D_2_O) LUMO+37 (and/or LUMO+38). These MOs show a quite delocalized shape attributed to important Rydberg and relevant σ_OH_^*^ antibonding character, therefore rendering special hydroxyl anion/deuteroxide formation. The formation of such anions may proceed through a curve crossing between the Rydberg and valence electronic states along the HO–H/DO–D coordinate. Note that the lowest-lying absorption band in the high-resolution VUV data of water established a Rydberg-valence mixing character, 3sa_1_/σ_OH_^*^, with a threshold at ∼6.5 eV and peaking at 7.464 eV.^57^ At higher energies, OH^–^/OD^–^ formation is due to the promotion of electrons to σ* antibonding like those obtained from LUMO+40 (and/or LUMO+44) and LUMO+46 (Table S3). These have been assigned in the energy loss spectra of Figure 2 to features at −8.86 ± 0.10 and −8.66 ± 0.20 eV (for H_2_O) and −12.36 ± 0.20 and −12.46 ± 0.50 eV (for D_2_O), respectively (Table 1), which are in good agreement with the DEA resonances of Fedor et al.^27^ at 8.6 (9.0) and 11.8 (12.0) eV, respectively. The fitting features that contribute to the maximum intensity in the energy loss spectrum at 14.5 ± 0.1 and 14.5 ± 0.3 eV for H_2_O and D_2_O, respectively, corresponding to vertical electron affinities of −10.16 ± 0.10 and −10.16 ± 0.30 eV, respectively, may be assigned to the broad nature of the OH^–^ and OD^–^^2^B_2_ resonances. The shape and charge distribution depicted in LUMO+45 (Figure S2) are indicative of a strong σ_OH_^*^/σ_OD_^*^ antibonding character.

A close inspection of H_2_O and D_2_O BRs reveals that above a 40 eV collision energy OD^–^ formation is slightly less effective than OH^–^ formation. This is not related to any D_2_O symmetry constraints given the identical electron energy loss spectrum with H_2_O^48^ but may be related to the nuclear dynamics within the TNI, which in the case of D_2_O renders a significant contribution to O^–^ formation. We do not have a plausible explanation for why that is not identical in H_2_O; however, autodetachment in electron transfer may be rather operative (considering the mass ratio of H and D atoms) than in DEA experiments. Although at higher collision energies K^+^ is less effective in relevant Coulomb interaction within the vicinity of the TNI, the MOs obtained with the K atom (from LUMO+48 to LUMO+51) show strong σ_O–H_^*^ and less pronounced σ_OH_^*^ antibonding character, with relevant electron density around the oxygen atom. This is clearly visible in the electron spin density of LUMO+51 (Figure S2).

From the appearance energy (AE) in the H_2_O energy loss spectrum (Figure 2) at Δ*E* ≈ 8.8 eV, one can obtain the HO–H bond dissociation energy (BDE) by taking the potassium ionization energy and the data from Table S4,^67^ i.e., *D*(HO–H) = AE(OH^–^) – IE(K) + EA(H). Thus, *D*(HO–H) = 5.21 ± 0.01 eV, which is in good agreement with the values of 5.15 eV (118.81 ± 0.07 kcal/mol)^68^ and 5.17 eV.^69^ Following the same approach for D_2_O, the energy loss spectrum shows a threshold feature at Δ*E* ≈ 9.0 eV. We obtain for the first time the DO–D bond dissociation energy [*D*(DO–D)] of 5.41 ± 0.01 eV, which is, as expected, slightly higher than in H_2_O given its higher boiling temperature under PTN conditions. In D_2_O, the DO–D energy value is higher than the O–D bond dissociation energy (5.176 eV^70^), which is consistent with that of its analogue H_2_O. Taking the values in Table S4 together with the BDE, we can obtain the enthalpies of formation from Δ_f_*H*_g_°(OH^–^) = *D*(H–OH) – EA(OH) = 3.34 eV and Δ_f_*H*_g_°(OD^–^) = *D*(D–OD) – EA(OD) = 3.58 eV. In the charge transfer process, if we add the potassium ionization energy, OH^–^ and OD^–^ are expected at 7.68 and 7.92 eV, respectively. The reaction thresholds were obtained assuming no excess energy (*E*^#^), yet the momentum conservation of the dissociating partners may impact the lighter fragment kinetic energy, thus shifting the energies to higher values. We note a difference of ∼1.1 eV from the energy loss data, which is certainly plausible given the kinetic energy release distribution of H^–^ in Figure S3.

The TOF mass spectra in the wide collision energy range investigated show O^–^ as the second most abundant fragment anion formed in charge transfer experiments from a neutral potassium atom to a neutral H_2_O/D_2_O molecule. From the BRs in Figure 2, the oxygen anion’s threshold is at ∼12.09 eV (H_2_O) and at ∼13.58 eV (D_2_O), increasing up to *E*_CM_ = 20 eV and contributing to ∼20% of the total anion yield. Above 22 eV, the yield modestly decreases to 40 eV, remaining constant regardless of the increasing energy in H_2_O, while showing a moderate enhancement in D_2_O. The lack of any discernible O^–^ signal below the threshold is due to the high OH^–^ yield at those energies. As the collision energy is increased, the MOs contributing to relevant antibonding character along the O–H bond (σ_O–H_^*^) are accessed (e.g., LUMO+46 and LUMO+47) and charge delocalization occurs mostly around the oxygen atom [e.g., see (LUMO+51) with a strong σ_O–H/O–D_^*^ antibonding character]. This may then contribute to the O^–^ yield yet compete with OH^–^ formation, despite the less pronounced σ_OH_^*^ antibonding character as noted above. For a thorough description of the underlying energetics of O^–^ formation, see the Supporting Information.

The BRs in Figure 1 show that H^–^/D^–^ is the less intense fragment ion in the TOF mass spectra, albeit a restricted low-energy region below ∼13 eV where it surpasses the O^–^ yield. We have noted that the collision-induced dissociation yielding preferentially OH^–^/OD^–^ relative to H^–^/D^–^ can be dictated by the higher electron affinity of OH. Thus, one would also expect a similar tendency for O^–^ formation given EA(O) ≈ 2 × EA(H/D) (Table S4). This seems not to be surprising given the relevant antibonding character along the O–H bond (σ_O–H_^*^) with the extra charge sitting on the higher-electron affinity radical. However, this is consistent with neither the experimental evidence nor the energetics of the product channels as shown in Table S2. This in turn may be related to the dynamics of the electron transfer process, which at such a low energy yields a collision time of >50 fs, i.e., a longer transit time of K^+^ in the proximity of the TNI, thus favoring H^–^/D^–^ formation via the ^2^B_1_ resonance. Note that O^–^ formation in electron transfer has been determined to proceed mainly through the ^2^A_1_ and ^2^B_2_ resonances, so relevant O–H antibonding character can be seen from the electron spin densities of LUMO+37 (and even LUMO+38), thus also providing a route for H^–^/D^–^ formation.

For collision energies above *E*_CM_ = 30 eV (Figure 1), the H^–^/D^–^ yield reaches <10% of the total anion yield and in D_2_O shows a tendency to reach 10%. This is due to the expense of the decrease in OD^–^ intensity. As the collision energy is increased, the contributions of MOs with relevant O–H/O–D antibonding character (σ_O–H_^*^/σ_O–D_^*^) are accessed (e.g., LUMO+46 and LUMO+47), and although charge delocalization mostly occurs around the oxygen atom, there is also some but not less significant density over the H/D atoms. It is well-established in ion-pair formation as the collision energy is well above the threshold of a particular fragment anion, and features in the K^+^ energy loss spectrum result from a vertical transition within the Franck–Condon region above the molecular ground state, resulting in an effective vertical electron affinity of the attained electronic state.^58^ A comprehensive description of the thermodynamic thresholds and the excess energy deposited in the unimolecular decomposition of the TNI via rovibrational energy distribution can be found in the Supporting Information.

We return to the energy loss features in Figure 2 that have not been discussed before and are assigned in Table 1. The H_2_O/D_2_O features at 18.5 ± 0.3/18.7 ± 0.4 eV, 20.3 ± 0.5/21.0 ± 0.6 eV, and 21.9 ± 0.3/23.0 ± 0.4 eV (Table 1) corresponding to vertical electron affinities of −14.16 ± 0.10/–14.36 ± 0.40 eV, −15.96 ± 0.50/–16.66 ± 0.60 eV, and −17.56 ± 0.30/–18.86 ± 0.40 eV, respectively, can also be assigned to excited electronic states converging to the different ionization energies, rendering Rydberg character for such MOs. Using the vertical ionization energies from experimental photoelectron spectroscopy data,^71,72^ the reasonable number of electronic states in the probed energy region, and the difficulty of performing an unambiguous assignment, features are assigned to Rydberg transitions of σ_OH_/σ_OD_ → (*n* + 1)/(*n* + 2)s, (*n* + 1)/(*n* + 2)p, (*n* + 1)/(*n* + 2)d character converging to 1b_2_^–1^ ionization energies of 18.55 and 18.66 eV for H_2_O and D_2_O, respectively (Table 1).

A careful inspection of the H_2_O energy loss spectrum in Figure 2 shows that features above 20 eV have low yields relative to those of the other electronic transitions. Although the calculated vertical energies of MOs and their natures are listed in Table 1, we are able to provide meaningful MOs for electronically excited states only when one occupied MO is replaced by another virtual (not occupied) MO. Nevertheless, the electron energy loss spectrum of H_2_O in coincidence with Lyman-α photon detection, at a 100 eV incident electron energy and an 8° electron scattering angle in the inner valence range, has been reported by Tsuchida et al.^73^ Hence, features with vertical excitation energies of −17.56 ± 0.30, −22.46 ± 0.40, and −27.66 ± 1.40 eV (Table 1) are tentatively assigned, on the basis of the features of Tsuchida et al.^73^ at 17.5, 24.2, and 27.9 eV, to singly excited (1b_2_^–1^) MO states and the latter two to doubly excited D1 and D2 states, respectively. Note that these authors assigned the underlying process of such transitions to neutral dissociation. Due to the particularly broad nature of the (1b_2_^–1^) MO (∼2 eV at full width at half-maximum),^73^ the K^+^ energy resolution, and uncertainty related to the Gaussian fittings in the energy loss spectrum, the feature at −19.56 ± 0.30 eV is assigned to be part of such a (1b_2_^–1^) molecular orbital state (Table 1).

Following a similar approach for D_2_O, we note that Kato et al.^74^ reported cross sections for Balmer-α fluorescence in the photoexcitation of H_2_O and D_2_O in the photon energy range of 17–41 eV. The superexcited states of Kato et al. yielding neutral dissociation at 19, 25, and 28 eV were assigned to single-hole one-electron states on the (1b_2_^–1^) ion state and doubly excited states D1 and D2, respectively.^74^ From the D_2_O energy loss spectrum, in general, we observe a reasonable agreement (within the experimental and fitting uncertainties) of the experimental vertical electron affinities at −21.86 ± 0.30, −24.76 ± 0.30, and −26.26 ± 0.20 eV with those of Kato et al.^74^ The isotope effect on the cross sections for the Balmer-α fluorescence in the photoexcitation of H_2_O/D_2_O was shown to be much more enhanced in the singly excited (1b_2_^–1^) MO states than in the doubly excited states D1 and D2; such experimental evidence was quantitatively discussed in terms of the state-resolved oscillator strengths in the fluorescence process.^74^ Moreover, the isotope effect is dependent on the survival probability related to the competition between autoionization (of the superexcited state) and bond excision into neutral fragments, and the probability of a molecule in that state to undergo fluorescence.^74^ Because the energy loss spectra in Figure 2 have arbitrary units, we can make a close comparison only between the most intense signal and the singly excited (1b_2_^–1^) MO states for each molecule. Ratios of ∼8% and ∼14% are obtained for H_2_O and D_2_O, respectively, thus suggesting that an isotope effect can be considered. As pointed out by Kato and co-workers,^74^ the current neutral potassium–neutral water molecule collision dynamics would also benefit from dedicated theoretical calculations on the potential energy surfaces and resonance widths of the superexcited states.

Here we report a novel electron transfer investigation in collisions of neutral K atoms with neutral H_2_O/D_2_O molecules in the laboratory energy range of 29–630 eV (lab frame). TOF mass spectra have been obtained in a wide collision energy range and allowed the assignment of fragment ions to OH^–^/OD^–^, O^–^, and H^–^/D^–^ with no evidence of parent anion formation. In contrast to dissociative electron attachment experiments in which H^–^ and D^–^ were reported to be the most intense fragment anions, the yields of OH^–^ and OD^–^ are predominant and account for ≳60% of the total anion yield. The branching ratios are energy dependent with a relevant noticeable isotope effect in the case of D_2_O relative to H_2_O. The different fragment anion thresholds of formation have been obtained and discussed on the basis of the underlying molecular mechanisms responsible for bond excision and mostly supported by quantum chemical calculations. Additionally, a kinetic energy release distribution for the hydrogen anion was obtained, thus revealing the role of statistical and direct dissociation in the collision process. Electronic state spectroscopy of H_2_O/D_2_O was thoroughly discussed from the experimental K^+^ energy loss spectra obtained, from which the DO–D bond dissociation energy has been determined for the first time to be 5.41 ± 0.10 eV. Finally, the information related to the collision dynamics revealed the role of the different resonances participating in the electron transfer process as well as the character of superexcited states, affording strong support for singly excited and doubly excited electronic states.

## Supporting Information Available

Introductory Note, experimental and theoretical methods, molecular optimized structure of K–H_2_O, shape of a selection of the molecular orbitals, O^–^ and H^–^/D^–^ reaction kinetics, H^–^ kinetic energy release distribution *D*(ε_d_), resonance positions, gas-phase reaction thresholds, calculated occupied (O) and virtual (V) molecular orbitals, and gas-phase standard heats of formation (Δ_f_*H*_g_°) and electron affinities (PDF)
Transparent Peer Review report available (PDF)
