# Correction to “Isotope Effect in D_2_O Negative Ion Formation in Electron Transfer Experiments: DO–D Bond Dissociation Energy”

**DOI:** 10.1021/acs.jpclett.3c02406

**Published:** 2023-10-02

**Authors:** Sarvesh Kumar, Masamitsu Hoshino, Boutheïna Kerkeni, Gustavo García, Paulo Limão-Vieira

In a recent Letter,^1^ a set of minor inconsistencies have been found in the main body text and in the Supporting Information files; these are now thoroughly corrected. Thus, we highlight the sections where these changes apply.

## Main Manuscript Body Text

Abstract: The DO–D bond dissociation energy has been reported to be 5.41 ± 0.10 eV, while considering EA(O) in the original manuscript. However, EA(OD) should be used instead, thus leading to a bond dissociation energy of 5.28 ± 0.20 eV.

Page 5365, left column, second paragraph; “From the appearance...” is reorganized and reads as follows:

Taking the values in Table S4 together with the BDE, the enthalpy of reaction can be obtained from Δ*H*_r_(OH^–^) = *D*(H–OH) – EA(OH) = 3.34 eV. In the charge transfer process, if we add the potassium ionization energy, OH^–^ is expected at 7.68 eV. The reactions threshold was obtained assuming no excess energy (*E*^#^), yet momentum conservation of the dissociating partners may impact on the lighter fragment kinetic energy, thus shifting the energy to a higher value. We note a difference of ∼1.1 eV for the energy loss data, which is certainly plausible given the kinetic-energy release distribution of H^–^ in Figure S3. Thus, from the appearance energy (AE) in the H_2_O energy loss spectrum (Figure 2) at Δ*E* ≈ 8.8 eV, one can obtain the HO–H bond dissociation energy (BDE) by taking the potassium ionization energy and the data from Table S4,^2^ i.e., *D*(HO–H) = AE(OH^–^) – IE(K) + EA(OH) – *E*^#^, yielding therefore *D*(HO–H) = 5.19 ± 0.20 eV which is in good agreement with the values 5.15 eV (118.81 ± 0.07 kcal/mol)^3^ and 5.17 eV.^4^ Following the same approach for D_2_O, the energy loss spectrum shows a threshold feature at Δ*E* ≈ 9.0 eV. We obtain for the first time the DO–D bond dissociation energy to be *D*(DO–D) = 5.28 ± 0.20 eV. In D_2_O the DO–D energy value is slightly higher than the O–D bond dissociation energy (5.176 eV^5^), which is in agreement with its analogue H_2_O.

Page 5366, right column, last paragraph; “Electronic state spectroscopy...” reads as follows:

The electronic state spectroscopy of H_2_O/D_2_O was thoroughly discussed from the experimental K^+^ energy loss spectra obtained, from which the DO–D bond dissociation energy has been obtained for the first time to be 5.28 ± 0.20 eV.

## Supporting Information

Enthalpies of formation (Δ_f_*H*_g_°) in reactions 2a.1–2b.2 and 4a.1–4b.2 are actually enthalpies of reaction (Δ*H*_r_). Table S2 decimal places for O^–^ and OH^–^ from H_2_O have been corrected; they now read 12.08 ± 0.20 and 7.80 ± 0.20.

## Supporting Information Available

Revised Supporting Information file (PDF)
