# Supplementary material for: Isotope Effect in D2O Negative Ion Formation in Electron Transfer Experiments: DO–D Bond Dissociation Energy
Source: J Phys Chem Lett. 2023 Jun 5;14(23):5362–9. doi: 10.1021/acs.jpclett.3c00786 (PMC10278136; doi:10.1021/acs.jpclett.3c00786)
Supplement: Supplementary file 1 — jz3c00786_si_001.pdf [file jz3c00786_si_001.pdf]

Isotope Effect in D_2O Negative Ion Formation in Electron Transfer Experiments: DO – D Bond Dissociation Energy

*Sarvesh Kumar,[1,ϕ] Masamitsu Hoshino,[2] Boutheïna Kerkeni,[3,4] Gustavo García,[5] and Paulo Limão-Vieira[1,]**

[1] Atomic and Molecular Collisions Laboratory, CEFITEC, Department of Physics, Universidade NOVA de Lisboa, 2829-516 Caparica, Portugal

[2] Department of Materials and Life Sciences, Sophia University, Tokyo, 102-8554 Japan

[3] ISAMM, Université de la Manouba, La Manouba 2010 Tunisia

[4] Département de Physique, LPMC, Faculté des Sciences de Tunis, Université de Tunis el Manar, Tunis 2092, Tunisia

[5] Instituto de Física Fundamental, Consejo Superior de Investigaciones Científicas (CSIC), Serrano 113-bis, 28006 Madrid, Spain

ϕSarvesh Kumar - Chemical Sciences Division, Lawrence Berkeley National Laboratory, One Cyclotron Road, Berkeley, 94720, California, USA

Introductory Note

The collision dynamics in electron transfer processes appears different from a free electron attachment process, meaning either different fragmentation patterns or fragment anions with different relative yields as to DEA processes.[1,2] In atom-molecule collisions, the charge transfer occurs when electrons follow adiabatically the nuclear motion in the vicinity of the crossing mediated by the covalent and ionic potential energy curves (and/or surfaces) involving the atomic projectile (K) and the molecular target (M).[2,3] Just for the case of simplicity, we take as an example a diatomic molecule (AB), where the ionic potential energy curve lies above the covalent. The energy separation at large atom-molecule distances is related to the endoergicity (ΔE) given by ΔE = IE(K) − EA(AB), with IE the ionization energy of the potassium (K) atom and EA the electron affinity of the target molecule. In case of an atom-molecule collision yielding ion-pair formation, $K + M \rightarrow (K^+ M^{-\#})$, the strong Coulomb interaction of $(K^+ M^{-\#})$

may delay autodetachment leading to a “stabilization” of the temporary negative ion (TNI), which can result in energy redistribution through the different available degrees of freedom leading either to a stable parent anion or different fragmentation channels. As far as potassium-water electron transfer process is concerned, in the unimolecular decomposition of the TNI, momentum transfer may provide OH^- considerable velocity to escape the collision complex, whilst in DEA, momentum transfer provides to H^- substantial velocity to escape the TNI. For further discussion, see main manuscript OH^- and H^- formation.

Experimental Method

The Lisbon laboratory equipped with a crossed molecular beam set up used to investigate the anionic fragmentation pattern of K collisions with H_2O and D_2O together with K^+ energy loss measurements, has been described elsewhere.[4–6] Briefly, it consists of two vacuum chambers both differentially pumped and interconnected by a gate valve with a 0.5 cm wide aperture, where the base pressure in the potassium chamber was 4×10^{-5} Pa and in the collision chamber was 5×10^{-5} Pa. The working pressure in the collision chamber after H_2O and D_2O effusion was 1×10^{-3} Pa (for K^+ energy loss measurements) and 5×10^{-4} Pa for (Time-of-Flight mass spectrometry). The neutral beam of potassium atoms is produced in the potassium chamber, where a commercial ion source (HeatWave, US) generates hyperthermal potassium cations (K^+_{hyp}) that are accelerated to a set kinetic energy towards the entrance of an oven. Here, these ions are resonantly charge exchanged in the charge exchange oven (CEO) with thermal potassium atoms (K^o_{th}), obtained by heating solid potassium at 393 K, yielding K^o_{hyp}. The resultant beam comprises K^+_{hyp} ions that did not charge exchange and are removed from the K^o_{hyp} beam by two deflecting plates placed at the exit of the CEO, before passing into the collision region. From the resonant charge-exchange process and the CEO slits apertures, the K^o_{hyp} beam is mainly composed of potassium atoms in the ground state configuration with its outermost electron as 4s. Thus, the experimental thresholds of formation are in assertion that K* in a 4p state would result in values at lower energies than those reported here (see Sec. III). Such has been shown in the past in other energy loss data from potassium collisions with pyrimidine,[5] halothane,[7] tetrachloromethane[6] and more recently with hexachlorobenzene[8,9] and nimorazole.[10] The K^o_{hyp} beam intensity is monitored at the entrance of the collision chamber by a surface ionization detector of the Langmuir-Taylor type. Thereafter, the K^o_{hyp} beam crosses at right angles with an effusive target beam, which is admitted to vacuum through a 1 mm diameter capillary from an external sample holder. In the collision region, the negative ions formed were extracted by a pulsed electrostatic field (380 Vcm^{-1}), and mass analysed by a

reflectron TOF spectrometer (r-TOF) with a mass resolution m/Δm ≈ 800. The beam energy resolution for TOF mass spectra collection in the collision energy range investigated was ~0.6 eV. The r-TOF mass calibration was performed from the well-known fragmentation patterns from collisions of potassium atoms with CH_3NO_2 and/or CCl_4 molecules.[6,11] Note that comprehensive background spectra (without the sample) were obtained and subtracted from the sample measurements. Branching ratios (BRs) for the fragment anions from H_2O and D_2O have been obtained and result from the fragment anion yield divided by the total anion yield at a given collision energy.

Potassium cations formed post-collision experiments were energy loss analysed in the forward scattering direction (θ ≈ 0°), while experiments were not performed in coincidence with r-TOF mass spectrometry. The analyser was operated in constant transmission mode, hence keeping the resolution constant throughout the entire scans. The estimated energy resolution during the experiments was ~1.2 ± 0.2 eV. The energy loss scale was calibrated using the K^+ beam profile from the potassium ion source serving as the *elastic* peak. H_2O and D_2O were supplied by Sigma-Aldrich with a stated purity 99.9% and were degassed through repeated freeze-pump-thaw cycles.

Theoretical Method

Electronic structure investigations of the molecular orbitals (MOs) formed in collisions between potassium (K) atoms and water (H_2O) have been performed to provide insight into the electron transfer process up to 30 eV. In particular, the analysis of the computed lowest unoccupied molecular orbitals (LUMOs) is crucial to assess the nature of the different electronic states that result in the detected negative ions of the current experiments.

The Minnesota hybrid-meta GGA functional M06-2X[12] family has been shown to be very sensitive to the integration grid employed and generally requires finer grids than other functionals in order to get reasonable numerical stability. Gaussian 16 automatically includes an ultrafine integration grid in the density functional theory (DFT) calculations in order to improve the accuracy of the results. The grid greatly enhances the accuracy at reasonable additional cost. This functional has been shown to be reliable in particular in computing energies.[13] Two gaussian basis sets have been used in our calculations, i.e., 6-311++G(2d,p), and 6-311++G(3df,3pd).[14,15]

The geometry of the K + H_2O was initially fully optimized at the M06-2X/6-311++g(2d,p) level of theory and the equilibrium distance between the potassium K and oxygen O atoms is 2.67 Å. The resulting optimized system upon the collision of K atoms with H_2O

resulted in the structure shown in Figure S1. All quantum chemical calculations have been performed with the Gaussian 16 program package.[16] The calculation has been carried out in Cartesian coordinates, with no symmetries. All electrons have been taken into account for potassium, oxygen and hydrogen atoms with the 6-311++g(2d,p) basis set during optimisations calculations. The natural molecular orbitals for K−H_2O have been calculated by M06-2X/6-311++g(3df,3pd) methodology.

O^- formation

We note that features resulting from the Gaussian fittings in the energy loss spectra of Figure 2 for H_2O and D_2O, peaking at 16.7 ± 0.2 eV and 16.8 ± 0.5 eV (Table 1), have thresholds at ~13 eV, and so can also be related to O^- formation. Note that in case of H_2O a difference of ~1.0 eV from the value obtained in the BRs is reasonable given the current energy resolution of the potassium beam. Notwithstanding, a close inspection of these features relative intensities to the maximum of the energy loss spectrum for each molecule, results in 0.44 for H_2O and 0.60 for D_2O. From a qualitative point of view, since we are not able to provide absolute cross-section values for reactions yielding the different anions', O^- formation must be more favourable in D_2O than in H_2O. This is in assertion with the relative difference between the OH^-/OD^- and the O^- yields (Figure 1), in particular above 40 eV collision energy.

In potassium-water/deuterium oxide collisions, formation of O^- may proceed through the following reactions:

$$K + H_2O \rightarrow (K^+H_2O^-) \rightarrow K^+ + (H_2O^-)^\# \rightarrow K^+ + O^- + H + H \qquad (1a.1)$$

$$K + H_2O \rightarrow (K^+H_2O^-) \rightarrow K^+ + (H_2O^-)^\# \rightarrow K^+ + O^- + H_2 \qquad (1a.2)$$

$$K + D_2O \rightarrow (K^+D_2O^-) \rightarrow K^+ + (D_2O^-)^\# \rightarrow K^+ + O^- + D + D \qquad (1b.1)$$

$$K + D_2O \rightarrow (K^+D_2O^-) \rightarrow K^+ + (D_2O^-)^\# \rightarrow K^+ + O^- + D_2 \qquad (1b.2)$$

where at least two O–H bonds must be broken yielding H + H (1a.1)/D + D (1b.1) or via other intramolecular reaction resulting in H_2 (1a.2)/D_2 (1b.2) formation. The enthalpies of formation $\Delta_f H_g^\circ$ (O^-) can be obtained as (Table S4):

$$\Delta_f H_g^\circ (O^-) = D(\text{H–OH}) + D(\text{O–H}) - \text{EA(O)} = 8.17\ \text{eV} \qquad (2a.1)$$

$$\Delta_f H_g^\circ (O^-) = D(\text{H–OH}) + D(\text{O–H}) - D(\text{H–H}) - \text{EA(O)} = 3.65\ \text{eV} \qquad (2a.2)$$

As far as D_2O is concerned, we shall use the bond dissociation energy from the present study to be D(D–OD) = 5.41 ± 0.10 eV, and $\Delta_f H_g^\circ$ (O^-) is now given by:

$$\Delta_f H_g^\circ (O^-) = D(D\text{–}OD) + D(O\text{–}D) - EA(O) = 9.15 \pm 0.10 \text{ eV} \quad (2b.1)$$

$$\Delta_f H_g^\circ (O^-) = D(D\text{–}OD) + D(O\text{–}D) - D(D\text{–}D) - EA(O) = 4.55 \pm 0.10 \text{ eV} \quad (2b.2)$$

Now adding 4.34 eV for the ionization energy of the potassium atom, reactions (1a.1) and (1b.1) proceed without an excess of energy (E^*), whereas reactions (1a.2) and (1b.2) appear to be quenched or at least with cross-sections values below the detection limit of our present TOF mass spectrometry experimental sensitivity.

The oxygen anion has been reported in DEA experiments to H_2O/D_2O with three resonances at 6.5 (7.0), 8.6 (9.0) and 11.8 (12.0) eV (Table S1) with increasing intensity as the electron energy is increased.[17] At its peak maximum in DEA experiments through the $^2B_1$ resonance, the cross section for O^- + H_2 production is ~ 40 times less intense than H^- + OH formation.[18] However, such experimental evidence is not consistent with the energetics of the product channels but rather with the dynamics of the DEA process.[19] Haxton *et al.*[19] have also reported that OH produced via the $^2B_1$ resonance is accompanied by extensive vibrational excitation. The oxygen anion produced via the $^2A_1$ resonance, was suggested to proceed predominantly through a three-body breakup process, O^- + H + H,[20,21] with such channel also reported for D_2O by Curtis and Walker.[22] Haxton *et al.*[23] reported that the $^2B_2$ resonance yielding H_2 + O^-, more energy goes into the rovibrational excitation of the H_2 fragment than into the kinetic energy of the recoil fragment. Fedor *et al.*[17] have reported mean values of kinetic-energy released for O^- from DEA experiments to H_2O/D_2O. These were obtained for the $^2B_1$ (6.4 eV), $^2A_1$ (8.4 eV) and $^2B_2$ (11.8 eV) resonances to be 0.12/0.14, 0.19/0.31 and 0.57/0.79 eV, respectively. Thus, this lends strong support to the experimental finding of O^- threshold at 12.09 eV in very good agreement with the expected value at 12.51 eV. Therefore, O^- formation in electron transfer processes may be attainable mainly through the $^2A_1$ and $^2B_2$ resonances (Table S2).

H^-/D^- formation

Formation of hydrogen/deuterium anions can proceed according to the reactions:

$$K + H_2O \rightarrow (K^+H_2O^-) \rightarrow K^+ + (H_2O^-)^\# \rightarrow K^+ + H^- + OH \quad (3a.1)$$

$$K + H_2O \rightarrow (K^+H_2O^-) \rightarrow K^+ + (H_2O^-)^\# \rightarrow K^+ + H^- + H + O \quad (3a.2)$$

$K + D_2O \rightarrow (K^+D_2O^-) \rightarrow K^+ + (D_2O^-)^\# \rightarrow K^+ + D^- + OD$ (3b.1)

$K + D_2O \rightarrow (K^+D_2O^-) \rightarrow K^+ + (D_2O^-)^\# \rightarrow K^+ + D^- + D + O$ (3b.2)

in DEA experiments, anions produced via reaction (3a.1) and (3b.1) have been reported to proceed dominantly via the $^2B_1$ resonance,[17,24–27] with a threshold at 4.35 eV.[27] The enthalpies of formation $\Delta_f H_g^\circ$ (H^-/D^-) can be obtained as (Table S4):

$\Delta_f H_g^\circ (H^-) = D(H–OH) – EA(H) = 4.41$ eV (4a.1)

$\Delta_f H_g^\circ (H^-) = D(H–OH) + D(O–H) – EA(H) = 8.85$ eV (4a.2)

$\Delta_f H_g^\circ (D^-) = D(D–OD) – EA(D) = 4.66 \pm 0.10$ eV (4b.1)

$\Delta_f H_g^\circ (D^-) = D(D–OD) + D(O–D) – EA(D) = 9.83 \pm 0.10$ eV (4b.2)

Now, adding the potassium ionization energy of 4.34 eV, the expected thresholds for reactions (3a.1) and (3b.1) are 8.75 (H_2O) and 9.00 eV (D_2O). These reactions have been assigned to the collision induced dissociation yielding H^- + OH ($X\ ^2\Pi$) and D^- + OD ($X\ ^2\Pi$) (Table S2). However, from the time-of-flight mass spectra of K + H_2O and K + D_2O collisions, such thresholds have been obtained at 10.24 and at 10.98 eV (Figure 1 and Table S2), thus meaning that H^-/D^- formation can proceed through the $^2A_1$ resonance. The energy difference can be attributed to the translational and internal energies of the fragments formed. A kinetic-energy release distribution (KERD) of hydrogen anion from H_2O (at 100 eV collision energy in the lab frame) has been obtained from a linear TOF mass spectrometer and is depicted in Figure S3.

The methodology used to obtain the KERD has been described elsewhere for different polyatomic molecular targets.[28–30] For further details on the methodology, the interested reader should consult Limão-Vieira *et al.*[28] and references therein. The H^- distribution shows a maximum at 0.28 ± 0.10 eV and the downward slope changes its character at ~1.7 eV, meaning that the excess energy of the TNI with respect to the HO–H^- dissociation level accounts for statistical and direct dissociation processes. Therefore, an excess energy of ≥ 1.7 eV can be attributed to translational energy, thus bringing the expected threshold of 8.75 eV close to the observed value of 10.24 eV. This is also expected to hold for D^- formation. Although we have not recorded any D^- profile from the linear TOF arrangement to extract the related KERD, the energy distributions at 8.5 eV electron energy obtained from the work of Belic *et al.*,[24] Fedor *et*

al.[17] and Ram *et al.*,[27,31] peak at ~1.7–2.0 eV thus giving us confidence about the expected excess energy value.

The distribution maximum at low kinetic-energy release is related to a statistical process where the excess energy is channelled into the available degrees of freedom of the OH radical. Note that OH formation via the $^2B_1$ resonance is accompanied by extensive vibrational[32,33] and rotational[32] excitations. However, at higher kinetic energy release, i.e., ≥ 1.7 eV, the distribution behaviour is indicative of direct dissociation, with the excess energy being channelled into translational energy of the two fragments. Therefore, for energies above 10.24 eV, reaction (3a.1) becomes accessible. This result is in good agreement with the threshold in the energy loss spectrum of H_2O (Figure 2) contributing to a feature with maximum intensity at 13.2 ± 0.1 eV, yielding a vertical electron affinity (VEA) of –8.86 ± 0.10 eV, in good agreement with the DEA resonance at 8.5–9.0 (Table S1). Regarding D_2O, the energy loss spectrum threshold is at ΔE ~ 11 eV corresponding to a vertical feature at 14.5 ± 0.3 eV, meaning a VEA of –10.16 ± 0.30 eV. Given the current potassium energy resolution of ~1 eV, and the related uncertainty to the fitting's procedure in Figure 2, we do not discard the possibility of the closest feature peaking at 13.0 ± 0.2 eV (Table II) to be a consequence of reaction (3b.1). Following the same rationale, reactions (3a.2) and (3b.2) have thresholds at 13.19 eV and 14.14 eV which can be related to the energy loss features peaking at 18.5 ± 0.3 and 18.7 ± 0.4 eV with vertical electron affinities of –14.16 ± 0.30 and –14.36 ± 0.40 eV (Figure 2 and Table 1).

Regarding the statistical process in the KERD of Figure S3, it was fitted with a function form Ref.[28] (and references therein) as:

$$D(\varepsilon_d) = C\left(1 - \frac{\varepsilon_d}{E_e}\right)^{s-2} \quad (5)$$

with ε_d the kinetic energy release, C a constant independent of the energy, s the adapted degree of freedom and E_e the available excess energy. The dashed line in Figure S3 was obtained for an excess energy of 4.1 eV from the difference between the resonance at 8.5 eV and the dissociation limit of the HO–H$^-$ configuration. The fitting procedure corresponds to s = 3 and $C = 4.0\times10^{-7}$, the latter with no physical meaning since the distribution is in arbitrary units. The value of three degrees of freedom seems appropriate as to those expected in water, yet the reaction path energy that is channelled into the accessible state in the collision process and to direct dissociation, is a quite complex intramolecular process within the TNI formed.

REFERENCES

(1) Illenberger, E.; Momigny, J. *Gaseous Molecular Ions. An Introduction to Elementary Processes Induced by Ionization*; H. Baumgärtel, E. U. Franck, W. G., Ed.; Steinkopff, Springer: Darmstadt, 1992.

(2) Kleyn, A. W.; Moutinho, A. M. C. Negative Ion Formation in Alkali-Atom – Molecule. *J. Phys. B At. Mol. Opt. Phys.* **2001**, *4075*, R1–R44.

(3) Kleyn, A.; Los, J.; Gislason, E. A. Vibronic Coupling At Intersections of Covalent and Ionic States. *Phys. Rep.* **1982**, *90*, 1–71.

(4) Almeida, D.; da Silva, F. F.; García, G.; Limão-Vieira, P. Dynamic of Negative Ions in Potassium-D-Ribose Collisions. *J. Chem. Phys.* **2013**, *139*, 114304.

(5) Mendes, M.; Pamplona, B.; Kumar, S.; da Silva, F. F.; Aguilar, A.; García, G.; Bacchus-Montabonel, M. C.; Limão-Vieira, P. Ion-Pair Formation in Neutral Potassium-Neutral Pyrimidine Collisions: Electron Transfer Experiments. *Front. Chem.* **2019**, *7*, 1–10.

(6) Regeta, K.; Kumar, S.; Cunha, T.; Mendes, M.; Lozano, A. I.; Pereira, P. J. S.; García, G.; Moutinho, A. M. C.; Bacchus-Montabonel, M. C.; Limão-Vieira, P. Combined Experimental and Theoretical Studies on Electron Transfer in Potassium Collisions with CCl4. *J. Phys. Chem. A* **2020**, *124*, 3220–3227.

(7) Lozano, A. I.; Maioli, L. S.; Pamplona, B.; Romero, J.; Mendes, M.; da Silva, F. F.; Kossoski, F.; Probst, M.; Süβ, D.; Bettega, M. H. F.; et al. Selective Bond Breaking of Halothane Induced by Electron Transfer in Potassium Collisions. *Phys. Chem. Chem. Phys.* **2020**, *22*, 23837–23846.

(8) Kumar, S.; Kilich, T.; Łabuda, M.; García, G.; Limão-Vieira, P. Anionic States of C6Cl6 Probed in Electron Transfer Experiments. *Phys. Chem. Chem. Phys.* **2022**, *24*, 366–374.

(9) Kumar, S.; Izadi, F.; Ončák, M.; Limão-Vieira, P.; Denifl, S. Hexachlorobenzene-Negative Ion Formation in Electron Attachment Experiments. *Phys. Chem. Chem. Phys.* **2022**, *24*, 13335–13342.

(10) Kumar, S.; Chouikha, I. B.; Kerkeni, B.; García, G.; Limão-Vieira, P. Enhanced Radiosensitisation of Nimorazole upon Charge Transfer. *Molecules* **2022**, *27*, 4134.

(11) Antunes, R.; Almeida, D.; Martins, G.; Mason, N. J.; Garcia, G.; Maneira, M. J. P.; Nunes, Y.; Limão-Vieira, P. Negative Ion Formation in Potassium–Nitromethane Collisions. *Phys. Chem. Chem. Phys.* **2010**, *12*, 12513–12519.

(12) Zhao, Y.; Truhlar, D. G. The M06 Suite of Density Functionals for Main Group Thermochemistry, Thermochemical Kinetics, Noncovalent Interactions, Excited States, and Transition Elements: Two New Functionals and Systematic Testing of Four M06-

Class Functionals and 12 Other Function. *Theor. Chem. Acc.* **2008**, *120*, 215–241.

(13) Peverati, R.; Truhlar, D. G. Quest for a Universal Density Functional: The Accuracy of Density Functionals across a Broad Spectrum of Databases in Chemistry and Physics. *Phil. Trans. R. Soc. A* **2014**, *372*, 20120476.

(14) Clark, T.; Chandrasekhar, J.; Spitznagel, G. W.; Schleyer, P. V. R. Efficient Diffuse Function-augmented Basis Sets for Anion Calculations. III. The 3-21+G Basis Set for First-row Elements, Li–F. *J. Comput. Chem.* **1983**, *4*, 294–301.

(15) Frisch, M. J.; Pople, J. A.; Binkley, J. S. Self-Consistent Molecular Orbital Methods 25. Supplementary Functions for Gaussian Basis Sets. *J. Chem. Phys.* **1984**, *80*, 3265–3269.

(16) Frisch, M. J.; Trucks, G. W.; Schlegel, H. B.; Scuseria, G. E.; Robb, M. A.; Cheeseman, J. R.; Scalmani, G.; Barone, V.; Petersson, G. A.; Nakatsuji, H.; et al. Gaussian 16 Rev. C.01, Wallingford, CT. Wallingford, CT 2016.

(17) Fedor, J.; Cicman, P.; Coupier, B.; Feil, S.; Winkler, M.; Głuch, K.; Husarik, J.; Jaksch, D.; Farizon, B.; Mason, N. J.; et al. Fragmentation of Transient Water Anions Following Low-Energy Electron Capture by H2O/D2O. *J. Phys. B At. Mol. Opt. Phys.* **2006**, *39*, 3935–3944.

(18) Haxton, D. J.; Zhang, Z.; McCurdy, C. W.; Rescigno, T. N. Complex Potential Surface for the 2B1 Metastable State of the Water Anion. *Phys. Rev. A* **2004**, *69*, 062713.

(19) Haxton, D. J.; Zhang, Z.; Meyer, H. D.; Rescigno, T. N.; McCurdy, C. W. Dynamics of Dissociative Attachment of Electrons to Water through the 2B1 Metastable State of the Anion. *Phys. Rev. A* **2004**, *69*, 062714.

(20) Adaniya, H.; Rudek, B.; Osipov, T.; Haxton, D. J.; Weber, T.; Rescigno, T. N.; McCurdy, C. W.; Belkacem, A. Imaging the Molecular Dynamics of Dissociative Electron Attachment to Water. *Phys. Rev. Lett.* **2009**, *103*, 233201.

(21) Haxton, D. J.; Rescigno, T. N.; McCurdy, C. W. Three-Body Breakup in Dissociative Electron Attachment to the Water Molecule. *Phys. Rev. A* **2008**, *78*, 040702.

(22) Curtis, M. G.; Walker, I. C. Dissociative Electron Attachment in Water and Methanol (5–14 EV). *J. Chem. Soc., Faraday Trans.* **1992**, *88* (19), 2805–2810.

(23) Haxton, D. J.; Rescigno, T. N.; McCurdy, C. W. Dissociative Electron Attachment to the H2 O Molecule. II. Nuclear Dynamics on Coupled Electronic Surfaces within the Local Complex Potential Model. *Phys. Rev. A* **2007**, *75*, 012711.

(24) Belic, D. S.; Landau, M.; Hall, R. I. Energy and Angular Dependence of H-(D-) Ions Produced by Dissociative Electron Attachment to H2O(D2O). *J. Phys. B At. Mol. Opt. Phys.* **1981**, *14*, 175–190.

(25) Compton, R. N.; Christophorou, L. G. Negative-Ion Formation in H2O and D2O. *Phys. Rev.* **1967**, *154*, 110–116.

(26) Melton, C. E. Cross Sections and Interpretation of Dissociative Attachment Reactions Producing OH-, O-, and H- in H2O. *J. Chem. Phys.* **1972**, *57*, 4218–4225.

(27) Ram, N. B.; Prabhudesai, V. S.; Krishnakumar, E. Resonances in Dissociative Electron Attachment to Water. *J. Phys. B At. Mol. Opt. Phys.* **2009**, *42*, 225203.

(28) Limão-Vieira, P.; Moutinho, A. M. C.; Los, J. Dissociative Ion-Pair Formation in Collisions of Fast Potassium Atoms with Benzene and Fluorobenzene. *J. Chem. Phys.* **2006**, *124*, 054306.

(29) Rebelo, A.; Cunha, T.; Mendes, M.; da Silva, F. F.; García, G.; Limão-Vieira, P. Kinetic-Energy Release Distributions of Fragment Anions from Collisions of Potassium Atoms with D-Ribose and Tetrahydrofuran. *Eur. Phys. J. D* **2016**, *70*, 130.

(30) Kumar, S.; Pereira, P. J. S.; García, G.; Limão-Vieira, P. Cl - Kinetic-Energy Release Distributions from Chlorobenzene and Related Molecules in Electron Transfer Experiments. *Eur. Phys. J. D* **2021**, *75*, 294.

(31) Ram, N. B.; Prabhudesai, V. S.; Krishnakumar, E. Dynamics of the Dissociative Electron Attachment in H2O and D2O: The A1 Resonance and Axial Recoil Approximationa. *J. Chem. Sci.* **2012**, *124*, 271–279.

(32) Trajmar, S.; Hall, R. I. Dissociative Electron Attachment in H2O and D2O: Energy and Angular Distribution of H- and D- Fragments. *J. Phys. B At. Mol. Opt. Phys.* **1974**, *7*, L458–L461.

(33) Haxton, D. J.; Adaniya, H.; Slaughter, D. S.; Rudek, B.; Osipov, T.; Weber, T.; Rescigno, T. N.; McCurdy, C. W.; Belkacem, A. Observation of the Dynamics Leading to a Conical Intersection in Dissociative Electron Attachment to Water. *Phys. Rev. A* **2011**, *84*, 1–4.

(34) NIST Chemistry WebBook, 2023. https://webbook.nist.gov/chemistry.

(35) Buchel'nikova, I. S. Cross Sections for the Capture of Slow Electrons by O2 and H2O Molecules of Halogen Compounds. *Sov. Phys. JETP* **1959**, *8*, 783–791.

(36) Fluendy, M. A. D.; Walker, I. C. Molecular Dynamics of Dissociative Electron Attachment in Water. *J. Chem. Soc., Faraday Trans.* **1995**, *91*, 2249–2255.

(37) Jungen, M.; Vogt, J.; Staemmler, V. Feshbach-Resonances and Dissociative Electron Attachment of H2O. *Chem. Phys.* **1979**, *37*, 49–55.

(38) Lozier, W. W. Negative Ions in Hydrogen and Water Vapor. *Phys. Rev.* **1930**, *36*, 1417–1418.

(39) Schulz, G. J. Excitation and Negative Ions in H2O. *J. Chem. Phys.* **1960**, *33*, 1661–1665.

(40) Ballard, R. E. The Electron Affinity of Water and the Structure of the Hydrated Electron. *Chem. Phys. Lett.* **1972**, *16*, 300–301.

(41) Kerr, J. A. Bond Dissociation Energies by Kinetic Methods. *Chem. Rev.* **1966**, *66*, 465–500.

(42) Bauschlicher, C. W.; Langhoff, S. R.; Walch, S. P. Theoretical Study of the Bond Dissociation Energies of Methanol. *J. Chem. Phys.* **1992**, *96*, 450–454.

(43) Herzberg, G. *Molecular Spectra and Molecular Structure I. Spectra of Diatomic Molecules*, 2nd ed.; Van Nostrand Reinhold Company: New York, 1950.

(44) Cheng, L. N.; Cheng, Y.; Yuan, K. J.; Guo, Q.; Wang, T.; Dai, D. X.; Yang, X. M. Photodissociation of HOD via the C̃1B1 State: OD/OH Branching Ratio and OD Bond Dissociation Energy. *Chin. J. Chem. Phys.* **2011**, *24*, 129–133.

(45) Ruscic, B.; Wagner, A. F.; Harding, L. B.; Asher, R. L.; Feller, D.; Dixon, D. A.; Peterson, K. A.; Song, Y.; Qian, X.; Ng, C. Y.; et al. On the Enthalpy of Formation of Hydroxyl Radical and Gas-Phase Bond Dissociation Energies of Water and Hydroxyl. *J. Phys. Chem. A* **2002**, *106*, 2727–2747.

Figures caption

Figure S1. Molecular optimized structure of K + H_2O collisional system, system K–O = 2.67 Å. K: purple, O: red, H: white.

Figure S2. Shape of a selection of the molecular orbitals (M06-2X/6-311++g(3df,3pd) for K + H_2O (K: magenta, O: red, H: white).

Figure S3. Kinetic-energy release distribution $D(\varepsilon_d)$ in collisions of K + H_2O obtained from the TOF mass spectrum of H^- at 100 eV in the lab frame. Statistical fitting added as a dashed line. The shape of the distribution does not change appreciably with the ±5% error bars, and these have not been added in the figure to avoid congesting it. The negative ion states are marked as arrows.

Tables caption

TABLE S1. Resonance positions and their assignments from dissociative electron attachment, electron transmission spectroscopy and electron scattering from H_2O and D_2O. See text for details (values in eV).

TABLE S2. Gas-phase reaction thresholds (ε_{th}) for anion formation compared with the present experiments (see text for details) and available data in the literature. Values in eV.

TABLE S3. Calculated occupied (O) and virtual (V) molecular orbitals of water at DFT/M062X/6-311++g(3df,3pd) level of theory.

TABLE S4. Gas-phase standard heats of formation ($\Delta_f H_g^{\circ}$) and electron affinities relevant in dissociative electron attachment to water, taken from Ref.[34] (see text for details).

Figure S1. Molecular optimized structure of K + H_2O collisional system, system K–O = 2.67 Å. K: purple, O: red, H: white.

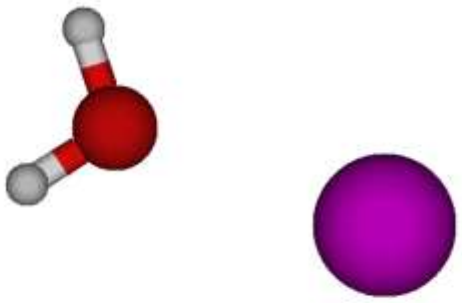

Figure S2. Shape of a selection of the molecular orbitals (M06-2X/6-311++g(3df,3pd) for K + H_2O (K: magenta, O: red, H: white).

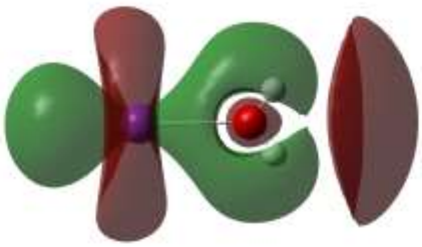	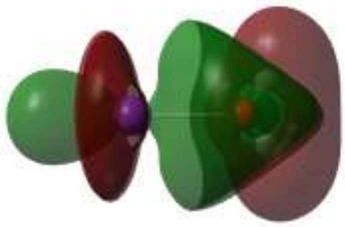
LUMO+33	LUMO+34
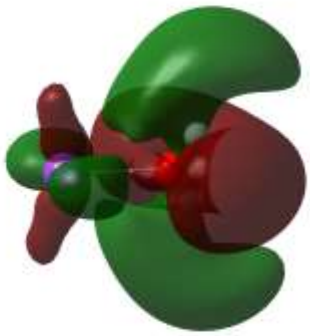	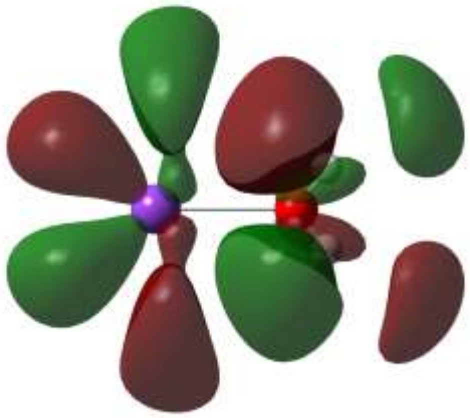
LUMO+37	LUMO+38

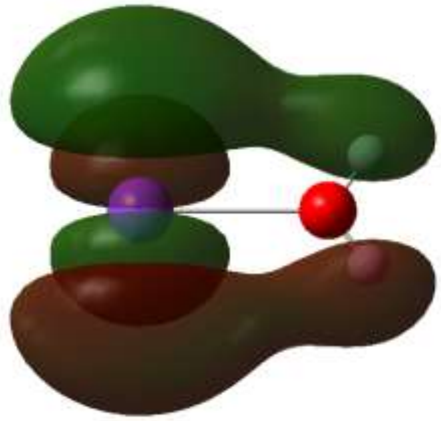	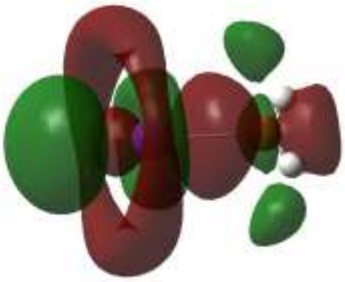
LUMO+40	LUMO+44
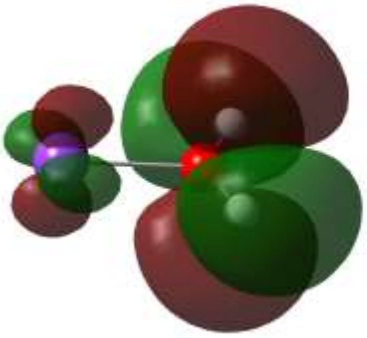	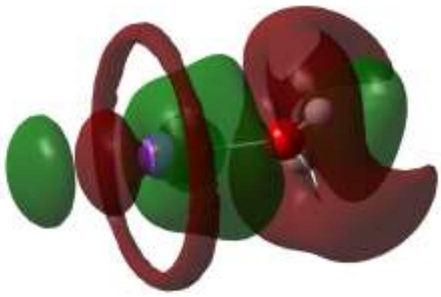
LUMO+45	LUMO+46
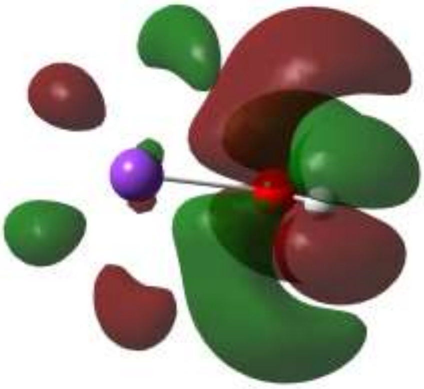	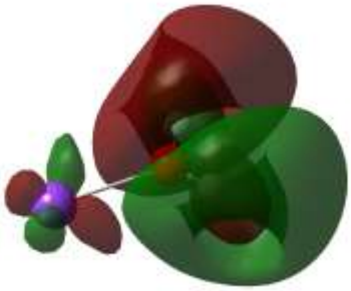
LUMO+47	LUMO+48

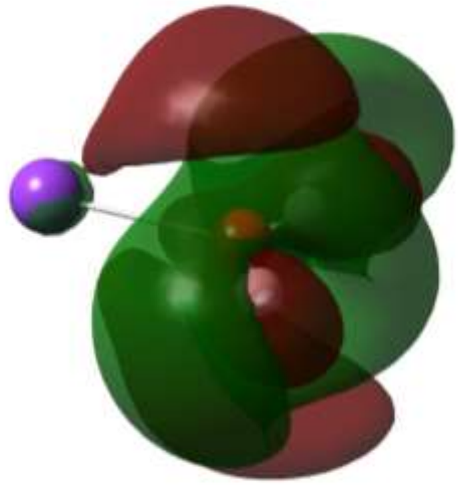	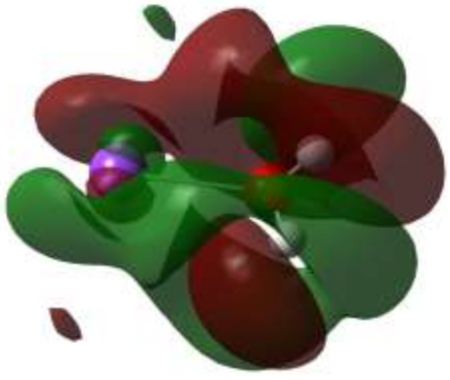
LUMO+49	LUMO+50
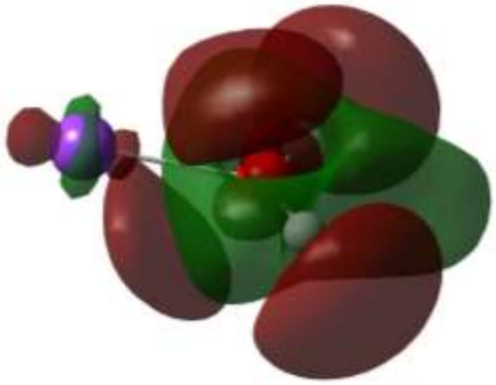	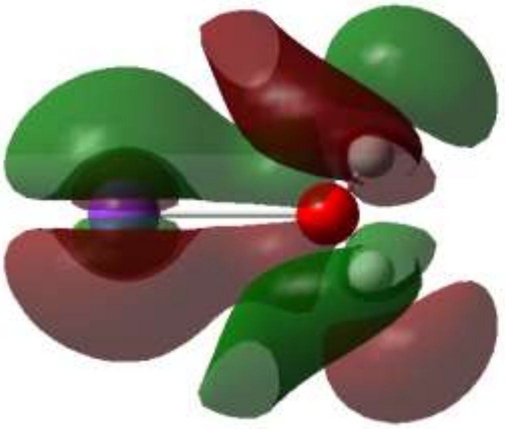
LUMO+51	LUMO+52
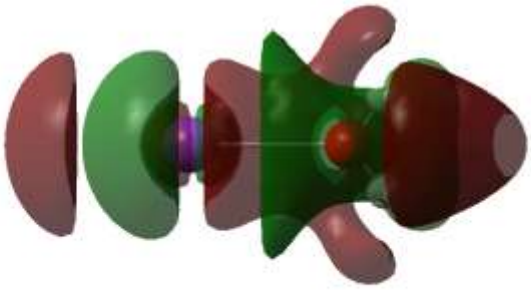	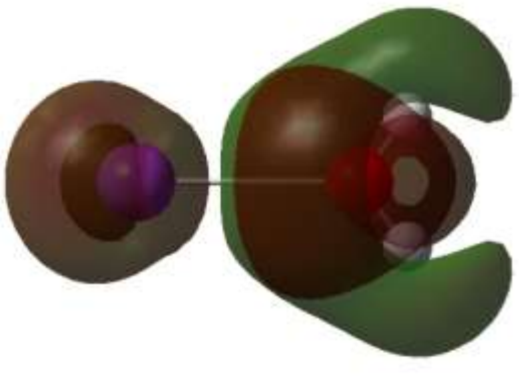
LUMO+54	LUMO+56

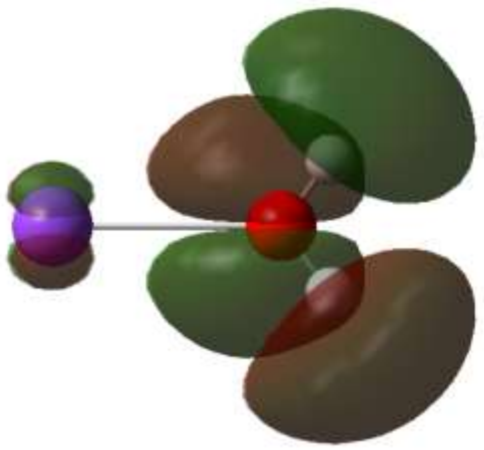	
LUMO+57	

Figure S3. Kinetic-energy release distribution $D(\varepsilon_d)$ in collisions of K + H_2O obtained from the TOF mass spectrum of H^- at 100 eV in the lab frame. Statistical fitting added as a dashed line. The shape of the distribution does not change appreciably with the ±5% error bars, and these have not been added in the figure to avoid congesting it. The negative ion states are marked as arrows.

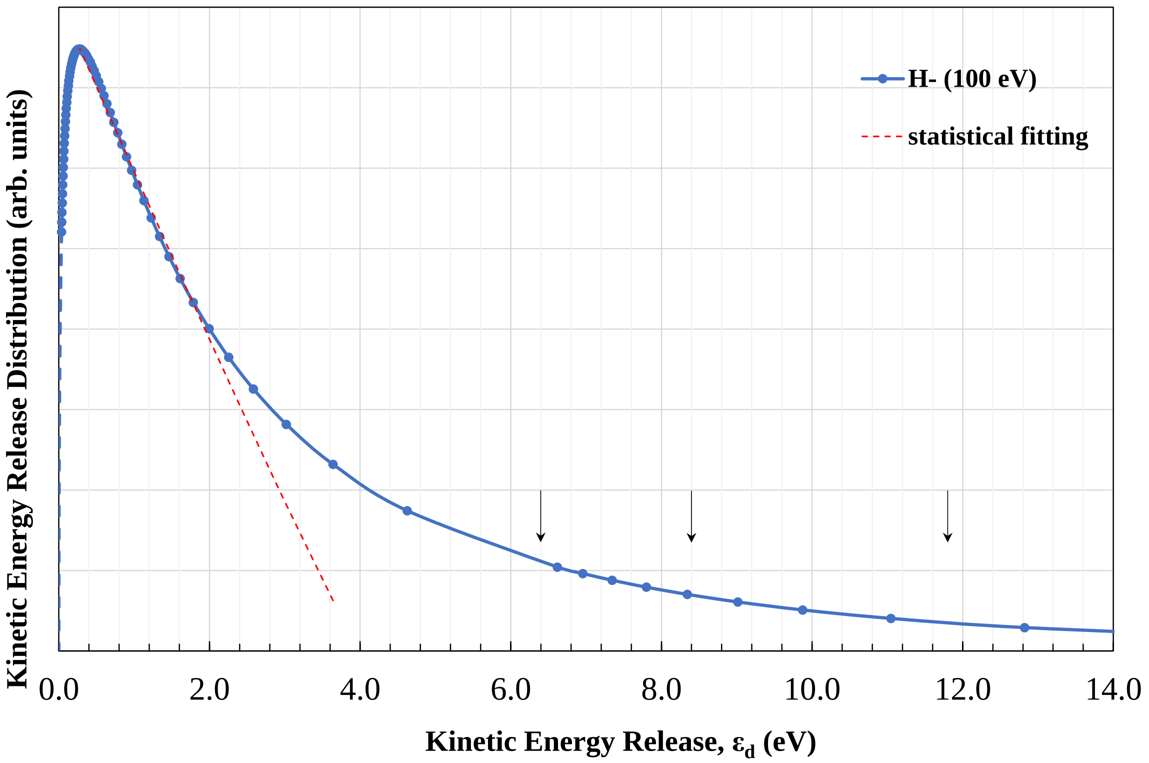

TABLE S1. Resonance positions and their assignments from dissociative electron attachment, electron transmission spectroscopy and electron scattering from H_2O and D_2O. See text for details (values in eV).

	Resonance positions (eV)					
	H_2O			D_2O		
Reference	H^-	O^-	OH^-	D^-	O^-	OD^-
Belić *et al.*[24]	6.5; 8.6; 11.8			6.5; 8.6; 11.8		
Buchel'nikova[35]	6.4; 8.6					
Compton and Christophorou[25]	6.5; 8.6	6.9; 8.9; 11.4		6.5; 8.6	7.0; 9.0; 11.8	
Curtis and Walker[22]				7.0; 9.5	6.5; 8.5; 11.0	
Fedor *et al.*[17]	6.4; 8.4	6.4; 8.4; 11.8	7.0; 8.5; 11.5	6.4; 8.4	6.4; 8.4; 11.8	7.0; 8.5; 11.5
Fluendy and Walker[36]				7.5; 9.5; 12.0	6.5; 8.8; 11.5	
Jungen *et al.*[37]		7.0; 9.1; 11.8	7.0; 9.1; 11.8		7.0; 9.1; 11.8	7.0; 9.1; 11.8
Lozier[38]	6.6; 8.8					
Melton[26]	6.4; 8.6	6.6; 8.4; 11.2	6.4; 8.4; 11.2			
Ram *et al.*[27]	6.5; 8.5; 11.8	7.0; 9.0; 12.0		6.5; 8.5; 11.8	7.0; 9.0; 12.0	
Schulz[39]	6.5; 8.8	12.0				
Trajmar and Hall[32]	6.5; 8.5			6.5		
Assignment (C_{2v})						
Refs.[24,36]	$^2B_1$; $^2A_1$; $^2B_2$			$^2B_1$; $^2A_1$; $^2B_2$		
Jungen *et al.*[37]		$^2B_1$; $^2A_1$; $^2B_2$	$^2B_1$; $^2A_1$; $^2B_2$			
Ram *et al.*[27]	$^2B_1$; $^2A_1$/$^2B_1$; $^2B_2$	$^2B_1$; $^2A_1$/$^2B_1$; $^2B_2$		$^2B_1$; $^2A_1$/$^2B_1$; $^2B_2$	$^2B_1$; $^2A_1$/$^2B_1$; $^2B_2$	

TABLE S2. Gas-phase reaction thresholds (ε_{th}) for anion formation compared with the present experiments (see text for details) and available data in the literature. Values in eV.

Resonance (eV)	H^-	ε_{th} (eV) calc.	ε_{th} (eV) this work	O^-	ε_{th} (eV) calc.	ε_{th} (eV) this work	OH^-	ε_{th} (eV) calc.	ε_{th} (eV) this work[#]	comment
6.5 (7.0)	H^- (^1S) + OH ($X\ ^2\Pi$)	4.41		O^- + H_2	3.65		OH^- + H	3.34	7.80 ± 0.10	OH vib. excited*; H_2 vib. excited
8.5 (9.0)	H^- + OH ($X\ ^2\Pi$)	4.41	10.24 ± 0.20	O^- + H_2	3.65					OH rot. excited**; H_2 vib. excited
				O^- + H + H	8.17	12.09 ± 0.20				
11.8 (12.0)	H^- + OH ($X\ ^2\Pi$)	4.41	10.24 ± 0.20	O^- + H + H	8.17	12.09 ± 0.20				OH vib. excited
	H^- (^1S) + OH* ($A\ ^2\Sigma$)	8.35[27]								OH rot. excited**
	H^- + H + O	8.85								
**Resonance (eV)**	**D^-**	**ε_{th} (eV) calc.**	**ε_{th} (eV) this work**	**O^-**	**ε_{th} (eV) calc.**	**ε_{th} (eV) this work**	**OD^-**	**ε_{th} (eV) calc.**	**ε_{th} (eV) this work[#]**	**Comment**
6.5 (7.0)	D^- (^1S) + OD ($X\ ^2\Pi$)	4.66		O^- + D_2	4.55		OD^- + D	3.58	8.30 ± 0.20	OD vib. excited*; D_2 vib. excited
8.5 (9.0)	D^- + OD ($X\ ^2\Pi$)	4.66	10.98 ± 0.20	O^- + D_2	4.55					OD rot. excited**
				O^- + D + D	9.15	13.58 ± 0.20				
11.8 (12.0)	D^- + OD ($X\ ^2\Pi$)	4.66	10.98 ± 0.20	O^- + D + D	9.15	13.58 ± 0.20				OD vib. excited
	D^- (^1S) + OD* ($A\ ^2\Sigma$)									OD rot. excited**
	D^- + D + O	9.83								

# from the energy loss data fitting

* also rotationally excited,[32] ** also modest vibrational excitation

TABLE S3. Calculated occupied (O) and virtual (V) molecular orbitals of water at DFT/M062X/6-311++g(3df,3pd) level of theory.

	Molecular orbitals	Energy (eV)
1	O	-3565.731787
2	O	-535.0006959
3	O	-369.3990272
4	O	-296.4168823
5	O	-296.4157122
6	O	-296.4078753
7	O	-40.3213355
8	O	-31.83437196
9	O	-21.98694726
10	O	-21.98052537
11	O	-21.98030768
12	O	-17.57957122
13	O	-14.07158638
14	O	-11.95339658
15	O	-3.121664597
16	V	-0.402511029
17	V	-0.280957705
18	V	-0.243405973
19	V	-0.147567422
20	V	0.20990874
21	V	0.22585462
22	V	0.292196013
23	V	1.10089161
24	V	1.535321611
25	V	2.354221482
26	V	2.713330328
27	V	2.750800426
28	V	2.849795499
29	V	2.856734406
30	V	2.881796106
31	V	3.065663535
32	V	3.217611993
33	V	4.24263822
34	V	5.597793151
35	V	5.745278939
36	V	6.112034188
37	V	6.213097328
38	V	7.851414088
39	V	8.492188135
40	V	8.943570838
41	V	9.213507926

42	V	9.414845075
43	V	9.417130832
44	V	9.437566594
45	V	10.69750884
46	V	11.70054825
47	V	13.59799917
48	V	13.69190572
49	V	15.71893732
50	V	16.65065566
51	V	19.61041243
52	V	20.47211583
53	V	20.6324454
54	V	22.46222157
55	V	23.15777216
56	V	25.26134944
57	V	27.46979945
58	V	27.63415631
59	V	27.81848633
60	V	28.99219565
61	V	30.05613418

TABLE S4. Gas-phase standard heats of formation ($\Delta_f H_g^\circ$) and electron affinities relevant in dissociative electron attachment to water, taken from Ref.[34] (see text for details).

Compound	$\Delta_f H_g^\circ$ (kJ mol^{-1})
H_2O	–241.826 ± 0.040
D_2O	–249.2
OH	38.99
OD	36.60
O	249.18 ± 0.10
H	218.00
D	221.72
O^-	108.3
	Electron affinity (eV)
H_2O	1.3[40]
OH	1.82767
OD	1.825543 ± 0.000044
O	1.439157 ± 0.000004
H	0.75497
D	0.754579 ± 0.000087
	Bond dissociation energy (eV)
O–H	4.436[41] (4.622[42], 4.35[43])
O–D	5.176[44]
H–OH	5.169[41] (5.152[45]; 5.453[42])
H–H	4.519[41] (4.478)
D–D	4.597[41] (4.556)
	Ionisation energy (eV)
OH	13.0170 ± 0.0002
OD	13.0290 ± 0.0002
O	13.61806
H	13.59844
D	13.603
K	4.34066 ± 0.00001
