# Supplementary material for: Isotope Effect in D2O Negative Ion Formation in Electron Transfer Experiments: DO–D Bond Dissociation Energy
Source: J Phys Chem Lett. 2023 Jun 5;14(23):5362–9. doi: 10.1021/acs.jpclett.3c00786 (PMC10278136; doi:10.1021/acs.jpclett.3c00786)
Supplement: Supplementary file 2 — jz3c00786_si_002.pdf [file jz3c00786_si_002.pdf]

jz-2023-007864.R1

Name: Peer Review Information for "Isotope Effect in D₂O Negative Ion Formation in Electron Transfer Experiments: DO – D Bond Dissociation Energy"

First Round of Reviewer Comments

Reviewer: 1

Comments to the Author
The authors have investigated scattering and electron transfer in K + H2O/D2O collisions. Much of the explanations are related to dissociative electron attachment processes detected in electron-H2O/D2O scattering experiments. Electron transfer is detected by measuring the negative ion time-of-flight mass spectra of OH–/OD–, O– and H /D from electron transfer at different collision energies with neutral potassium atoms. From the incident K energy dependence of the branching ratios, they find a strong isotopic effect in D2O. They also recorded potassium cation energy loss spectra in the forward scattering direction at 205 eV impact energy (lab frame). The spectra served to identify the various electron exchange processes with the help of quantum chemical calculations for the lowest-lying unoccupied molecular orbitals in the presence of a potassium atom. The DO–D bond dissociation energy is reported for the first time (5.41 ± 0.10 eV). Furthermore, they determine that the lowest-lying triplet states of H2O and D2O lie at −4.76 ± 0.30 and −5.16 ± 0.30 eV.

These results have been produced by a group of experts in atom-molecule collisions and electron-molecule collision processes. As shown in this letter, this combined expertise places them in a good position to explain such processes occurring between energetic K atoms and water molecules. To my knowledge, this is the most complete data available so far on such collisions, and for the first time, all know dissociative electron attachment, electronic excitation and dissociation processes are merged to explain the mass spectra in combination with the K+ energy-loss data. Furthermore, their identification of the quantum states involved are supplemented by calculations for the lowest-lying unoccupied molecular orbitals in the presence of a potassium atom. In other words, this work is very complete and stands as a significant advance in our understanding of the dynamics of neutral-atom-water scattering and corresponding electron transfer reactions. The results are sound, well described and the explanations are plausible. The conclusions are significant and logical consequences of their results. The data is well explained in terms of electron affinities and excitation energies. I am impressed by the quality and relevance of this work. I believe their paper should be accepted for publication in JPCL, after consideration of my suggestions below.

In this scattering problem, the time of collision between K and water is of the order or longer that the lifetime of the transient anion formed by electron transfer and its dissociation. In my view, the intermediate state is K electrostatically bound to a water molecule during the collision time. Therefore, the main changes in the intensities of the anion radicals produced by electron transfer would be very different from those of DEA, because in the electron transfer case the dissociating fragments arise from a compound target made of a water molecule plus a neutral K, both being temporarily electrostatically bound together during the collision time. In this case, momentum transfer provides to OH- considerable velocity to escape the collision complex, whereas in DEA, momentum transfer provides to H- considerable velocity to escape de H2O- anion. This consideration should at least partially explain why, in K + H2O/D2O collisions, the yield of OH- is more intense than H-. I am surprised this is not mentioned and wonder if I simply misunderstood or missed some explanations.

Further comments are following based on the suggestions of the editors.

1. What is the major advance reported in the paper?

The obvious major advance is a very elaborate description of electron transfer in K + H2O/D2O collisions and the spectroscopic consequences. However, it could be more significant by explaining in the introduction that the description of electron transfer processes in the formation of transient anions of water is important to explain the production of radicals in radiobiology, and that the present experiments provide a method to envision electron attachment to H2O when it is bound strongly to other cell constituents. In the cell, like in the temporary K+ H2O/D2O collision complex, the DEA does not proceed on isolated water but often on a complex containing water (e.g., structural water in DNA). Obviously, the present method may be adequate to understand DEA in condensed matter. The introduction does not explain well the relationship between the present experiments and radiobiological damage. The first paragraph gives a general view of electron induced processes in biologically relevant molecules that have been central to assess the underlying molecular mechanisms responsible for bond excision and chemical modification, after interaction of primary radiation with living tissue; this is an appropriate general introduction. However, the following paragraph does not provide a tangible link between the present measurements and radiation damage in biological tissue. A sentence like "Also relevant to the scope of this work are quadrupole mass spectrometry studies of negative and positive ions formed in 1–4 keV H–, O–, and OH– collisions with water molecules" is too general. The authors should elaborate more on their statement "In the unimolecular decomposition of the temporary negative ion formed after electron capture, the sort of fragmentation and the relative yields that are attainable in electron transfer processes, may differ from DEA experiments" and explain why the information obtained from formation of transient anions by electron transfer is relevant to radiobiological damage.

2. What is the immediate significance of this advance?

Better comprehension of electron transfer processes in collisions between water and atoms of low ionization potential. Determination of the DO–D bond dissociation energy, and character of the singly excited molecular orbital and doubly excited states of water.

3. Technical suggestions.

The experiments were performed with state-of-the-art techniques. I have no suggestion.

Reviewer: 2

Comments to the Author

Report on "Isotopic Effect in D_2O Negative Ion Formation in Electron Transfer Experiments: DO – D Bond Dissociation Energy" by Sarvesh Kumar et al., submitted to JPC Letters.

The authors report on a new type of isotope effect which arises in an electron transfer from potassium atoms to H_2O/D_2O. In recent years, the electron transfer experiments from the Lisbon group have been crucial in amending the (much more abundant) data on electron collisions with molecules and thus expanding our knowledge on the electron-induced chemistry. Here, twofold experimental information is presented: (i) anion mass spectra upon potassium collisions (fragmentation patterns and branching

ratios) and (ii) potassium cation energy loss spectra. The first one reveals the surprising isotope effect in the branching ratios. I find the interpretation of this effect to be plausible. Any such new information about a molecule as fundamental as water represents a major advance and is of immediate significance. Normally, I would thus recommend the paper for JPCL right away. However, there is one major problem.

The problem concerns the K^+ energy loss spectra in figure 2. The lowest band in H_2O has a maximum of 9.1 eV which corresponds to "vertical electron affinity" of -4.76 eV. The authors assign this feature to the $^3B_1$ state of H_2O and state that "is in excellent agreement with the electron impact excitation value of 4.5 eV [69, 70]." They then call for further investigation of the disagreement with high-level theory (predicting this state at 7.13 eV). The triplet state energy also made it to the abstract and is thus one of the selling points of the paper.

References 69 and 70 are the 1971 and 1973 papers from the group of S. Trajmar, who did this assignment based on a 4.5 eV band in the electron energy loss spectra (EELS) of H_2O. However, already in 1975, the same group re-assigned this band and postulated the energy of the lowest triplet state to be 7.0 eV (Chutjian et al. http://dx.doi.org/10.1063/1.431370, current reference 47). The 4.5 eV band which (occasionally) appeared in EELS had been a puzzle until a paper of Edmonson et al. https://doi.org/10.1063/1.436768 who postulated that it originates from a background-scattered electrons in a water-contaminated apparatus (by the way, the work of Edmonson is the first ion energy loss spectrum of H_2O and should be definitely mentioned in the present paper).

To resolve the question of possible position of $^3B_1$ between 4-6 eV, I now recorded the EELS spectrum of H_2O at conditions favoring excitation of triplet states (low residual energy, high scattering angle).

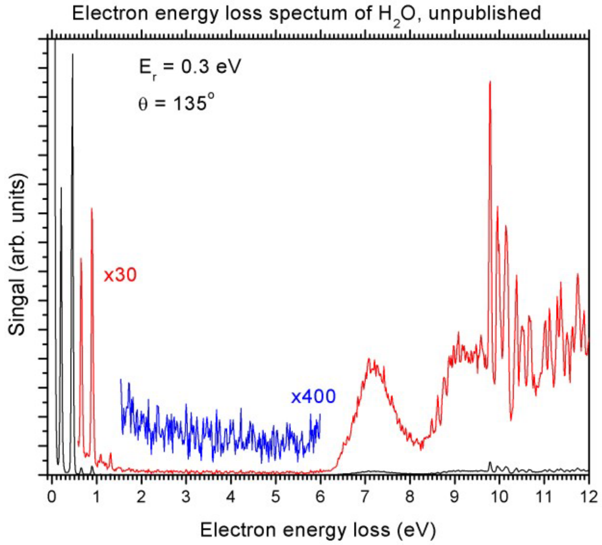

Clearly, there is no detectable signal below the onset of the 7 eV band. The $^3B_1$ experimental energy is thus at 7.0 eV, in an agreement with Chutjian et al., and with good agreement with the theory.

At the same time, the K^+ energy loss spectrum in figure 2 clearly shows the 9.1 eV band. It cannot be the excitation of the lowest triplet state, nor the spurious processes seen by Edmonton et al. since those are unique for electron scattering. If this peak is an experimental artifact, this creates worries about the reliability of the rest of the K^+ energy loss spectrum. If this peak is real, a different interpretation should be put forward.

Author's Response to Peer Review Comments:

Manuscript ID: jz-2023-007864

Lisbon, 19 May 2023

Editor for The Journal of Physical Chemistry Letters

Dear [redacted]

We submit a revised version of the manuscript for your consideration, where the accurate denomination requires replacing "Isotopic" by "Isotope", thus reading "Isotope Effect in D_2O Negative Ion Formation in Electron Transfer Experiments: DO – D Bond Dissociation Energy". We hope that this small correction in the title does not constitute any constraint to the editorial office as far as submission is concerned.

We acknowledge the reviewers for their perceptive and constructive comments and hope that the changes made to the manuscript are to their satisfaction. A reply to the reviewers' comments is enclosed and changes to the manuscript are highlighted in yellow colour for easier and faster tracking.

Yours sincerely,

Paulo Limão-Vieira on behalf of all the authors

Reviewer: #1

In this scattering problem, the time of collision between K and water is of the order or longer that the lifetime of the transient anion formed by electron transfer and its dissociation. In my view, the intermediate state is K electrostatically bound to a water molecule during the collision time. Therefore, the main changes in the intensities of the anion radicals produced by electron transfer would be very different from those of DEA, because in the electron transfer case the dissociating fragments arise from a compound target made of a water molecule plus a neutral K, both being temporarily electrostatically bound together during the collision time. In this case, momentum transfer provides to OH^- considerable velocity to escape the collision complex, whereas in DEA, momentum transfer provides to H- considerable velocity to escape de H_2O^- anion. This consideration should at least partially explain why, in K + H_2O/D_2O collisions, the yield of OH^- is more intense than H^-. I am surprised this is not mentioned and wonder if I simply misunderstood or missed some explanations.

Authors' reply: this is certainly true. Given the space limitation imposed to authors by the journal within the main body text, a paragraph accounting this rationale has been added to Supplementary Information as an Introductory note section.

The introduction does not explain well the relationship between the present experiments and radiobiological damage. The first paragraph gives a general view of electron induced processes in biologically relevant molecules that have been central to assess the underlying molecular mechanisms responsible for bond excision and chemical modification, after interaction of primary radiation with living tissue; this is an appropriate general introduction.

However, the following paragraph does not provide a tangible link between the present measurements and radiation damage in biological tissue. A sentence like "Also relevant to the scope of this work are quadrupole mass spectrometry studies of negative and positive ions formed in 1–4 keV H–, O–, and OH– collisions with water molecules" is too general. The authors should elaborate more on their statement "In the unimolecular decomposition of the temporary negative ion formed after electron capture, the sort of fragmentation and the relative yields that are attainable in electron transfer processes, may differ from DEA experiments" and explain why the information obtained from formation of transient anions by electron transfer is relevant to radiobiological damage.

Authors' reply: as far as the first half of the comment is concerned we have followed the reviewer's concern and adjusted the paragraph accordingly. The note on the electron transfer studies with high-energy projectiles H^-, O^-, and OH^- with water, has been properly changed. For the second half comments from the reviewer, these have been properly addressed in Supplementary Information - Introductory note section as noted in the response above.

Reviewer: #2

The 4.5 eV band which (occasionally) appeared in EELS had been a puzzle until a paper of Edmonson et al. https://doi.org/10.1063/1.436768 who postulated that it originates from a background-scattered electrons in a water-contaminated apparatus (by the way, the work of Edmonson is the first ion energy loss spectrum of H2O and should be definitely mentioned in the present paper).

Authors' reply: as suggested by the reviewer, Edmonson, Lee and Doering (J. Chem. Phys. 69 (1978) 1445) reference has been added accordingly.

To resolve the question of possible position of $^3B_1$ between 4-6 eV, I now recorded the EELS spectrum of H_2O at conditions favoring excitation of triplet states (low residual energy, high scattering angle). Clearly, there is no detectable signal below the onset of the 7 eV band. The $^3B_1$ experimental energy is thus at 7.0 eV, in an agreement with Chutjian et al., and with good agreement with the theory. At the same time, the K^+ energy loss spectrum in figure 2 clearly shows the 9.1 eV band. It cannot be the excitation of the lowest triplet state, nor the spurious processes seen by Edmonton et al. since those are unique for electron scattering. If this peak is an experimental artifact, this creates worries about the reliability of the rest of the K^+ energy loss spectrum. If this peak is real, a different interpretation should be put forward.

Authors' reply: we are very thankful to the reviewer for alerting on Edmonson et al.'s publication back in 1978, which somehow we have missed during the literature survey, as well as his/her effort in recording an electron energy loss spectrum under non-dipolar conditions to reinforce the rationale about the absence of a triplet state at the energy position we initially assigned to.

We have re-analysed the energy loss data and the procedure related to the background subtraction from the sample's spectrum. Recording an energy loss spectrum of water and accounting for its contribution as a background contaminant, that is always present in vacuum chambers, was not performed accordingly. A recorded background energy loss spectrum in Figure 1 below, before correction to the analyser's transmission, clearly shows that the only visible feature at ~ 9 eV is solely due to water background contribution, where no other discernible contributions appear in the spectrum.

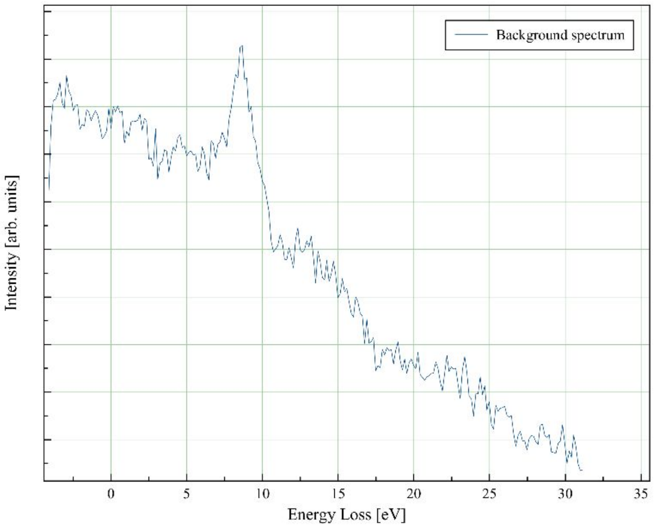

Figure 1. Energy loss spectrum of background at potassium collision energy of 205 eV in the laboratory frame.

A careful inspection of the energy loss spectra of H_2O and D_2O reveals that features at ~9 eV (for H_2O and D_2O) cannot proceed from the sample measurement but from the background as

suggested by the reviewer. Again, we appreciate his/her expertise to alert on such possible contamination. Notwithstanding, we have re-assessed the energy loss spectra of H_2O and D_2O, and found no appreciable change to the different fitting positions (to within the experimental uncertainty) from which we extract the vertical excitation energies (Table 1 in the manuscript). Thus, the consistency of the contributions related to H_2O and D_2O electronic state spectroscopy, is not affected.

As far as authors are concerned, the present contribution still addresses relevant achievements reported for the first time in potassium-water molecule collision experiments as to:

1. the relevant isotope effect in D_2O relative to H_2O;
2. the experimental DO–D bond dissociation energy;
3. the role of singly excited and doubly excited states probed by the collision dynamics in such atom-molecule collisions.
